# Dynamics of in silico leukocyte rolling, activation, and adhesion

**DOI:** 10.1186/1752-0509-1-14

**Published:** 2007-02-19

**Authors:** Jonathan Tang, Klaus F Ley, C Anthony Hunt

**Affiliations:** 1 The UCSF/UCB Joint Graduate Group in Bioengineering, University of California, Berkeley, CA, USA; 2 Robert M. Berne Cardiovascular Research Center and Departments of Biomedical Engineering, Molecular Physiology and Biological Physics, University of Virginia, Charlottesville, VA, USA; 3 The Department of Biopharmaceutical Sciences, Biosystems Group, University of California, San Francisco, CA, USA

## Abstract

**Background:**

We present a multilevel, agent based, in silico model that represents the dynamics of rolling, activation, and adhesion of individual leukocytes in vitro. Object-oriented software components were designed, verified, plugged together, and then operated in ways that represent the molecular and cellular mechanisms believed responsible for leukocyte rolling and adhesion. The result is an in silico analogue of an experimental in vitro system. The experimentally measured, phenotypic attributes of the analogue were compared and contrasted to those of leukocytes in vitro from three different experimental conditions.

**Results:**

The individual in silico dynamics of "rolling" on simulated P-selectin, and separately on simulated VCAM-1, were an acceptable match to individual in vitro distance-time and velocity-time measurements. The analogues are also able to represent the transition from rolling to adhesion on P-selectin and VCAM-1 in the presence of GRO-α chemokine. The individual in silico and in vitro behavioral similarities translated successfully to population level measures. These behavioral similarities were enabled in part by subdividing the functionality of the analogue's surface into 600 independent, "cell"-controlled, equally capable modules of comparable functionality.

**Conclusion:**

The overlap in phenotypic attributes of our analogue with those of leukocytes in vitro confirm the considerable potential of our model for studying the key events that determine the behavioral outcome of individual leukocytes during rolling, activation, and adhesion. Our results provide an important foundation and framework for future in silico research into plausible causal links between well-documented, subcellular molecular level events and the variety of systemic phenotypic attributes that distinguish normal leukocyte adhesion from abnormal disease-associated adhesion.

## Background

What molecular-level events determine the behavioral outcome of individual leukocytes during rolling, activation, and adhesion to venular surfaces? These processes are necessary steps for the proper recruitment of leukocytes from circulating blood to sites of inflammation. Once at the target site, leukocytes help destroy pathogens and decompose damaged tissue. However, inflammatory mechanisms are also associated with diseases such as asthma, rheumatoid arthritis, multiple sclerosis, and atherosclerosis. Such diseases can be characterized by inappropriate leukocyte recruitment and the misdirected actions of leukocytes towards healthy host-tissue [1].

Rolling and adhesion following attachment are two of the least complicated of many individual leukocyte behaviors that have been studied in vitro using flow chamber assays. Those behaviors are by no means deterministic. A striking feature of such studies is that cell behavior is heterogeneous: individual cell behaviors under identical conditions can be quite different. No two cells behave the same, yet collective behaviors are robust and fall reliably within narrow ranges. Rolling, for example, exhibits an irregular, jerky stop-and-go pattern along with highly fluctuating rolling velocities [2]. Additionally, in the presence of chemokine, only a fraction of a leukocyte population will adhere firmly [3].

A goal in systems biology research is to understand linkages from molecular level events to system phenotype: link genotype to phenotype. That task requires having plausible, adequately detailed design plans for how components (single and composite) at various system levels are thought to fit and function together. Ideas about such plans can be induced from the results of experiments. Experimentation is then used to reconcile different design plan hypotheses. More is needed, however, to actually demonstrate that a design plan is functionally plausible, which is very different from demonstrating that it is consistent with measured behaviors. The former requires that one assemble individual components according to a design, and then show that the constructed device, an analogue – on its own – exhibits behaviors that match those observed in the original experiment.

Building such analogues in silico is now feasible. To make it practicable, we need multilevel modeling and simulation methods that make it easy to test, reject, and refine many candidate design plans. In this report, we describe discovering, building, and testing aspects of a simple yet plausible design plan. That achievement is an essential step toward the long-term biological goal for this project: develop scientifically useful, validated simulations of leukocyte recruitment under physiological and pathophysiological conditions. We use the synthetic modeling approach. Object-oriented software components were designed, verified, plugged together, and then operated in ways that represent the mechanisms and processes believed responsible for leukocyte rolling and adhesion. The result is an analogue of an in vitro experimental system. An analogue refinement method is used in which experimentally measured phenotypic attributes are iteratively compared and contrasted to those of leukocytes in vitro.

During leukocyte rolling, the initial interactions between leukocyte and surface are primarily mediated by the selectin family of receptors and their respective carbohydrate ligands found on the membranes of both the leukocytes and endothelial cells. Transient selectin-ligand interactions enable rolling. Leukocyte arrest is mediated by the integrin receptor family. Integrins exist largely in nonadhesive states to prevent leukocytes from sticking non-specifically to blood vessels. As leukocytes roll along the venular surface, they use their chemokine receptors to detect immobilized chemokines on activated endothelial cells. Upon detection, intracellular signaling events trigger integrin conformational changes, increasing integrin-ligand affinity. High affinity integrins enable the leukocytes to firmly adhere to the vessel wall or flow chamber surface [4]. Signaling through chemokine receptors can also induce lateral movement and clustering of integrins within the membrane to promote leukocyte adhesion [5,6]. In addition, the engagement of some selectins and integrins with their ligands can initiate various activation signals to enhance adhesion [7,8].

With their Adhesive Dynamics simulations, Hammer and co-workers have elegantly demonstrated how challenging it can be to generate similar rolling characteristics in silico when using physical and mechanical representations combined with traditional modeling formalisms [9-11]. In their models, leukocytes are represented as solid spheres decorated with rod-like "microvilli" containing receptors at their tips. Using a Monte Carlo algorithm for the determination of receptor-ligand interactions, they have successfully produced a jerky stop-and-go pattern similar to that observed for rolling leukocytes. Such models can be fragile to context. When they target specific phenomena and constrain their use to account for specific sets of data, it becomes difficult to extend the model to different experimental circumstances or to help explain different phenomena or particular examples of individual behavior. It becomes difficult to explore potential mechanistic differences between individual cell behaviors, for example. Are there alternative modeling and simulation approaches that are less susceptible to such problems? The multilevel approach described here was selected in part to help circumvent those problems.

**Figure 1:**
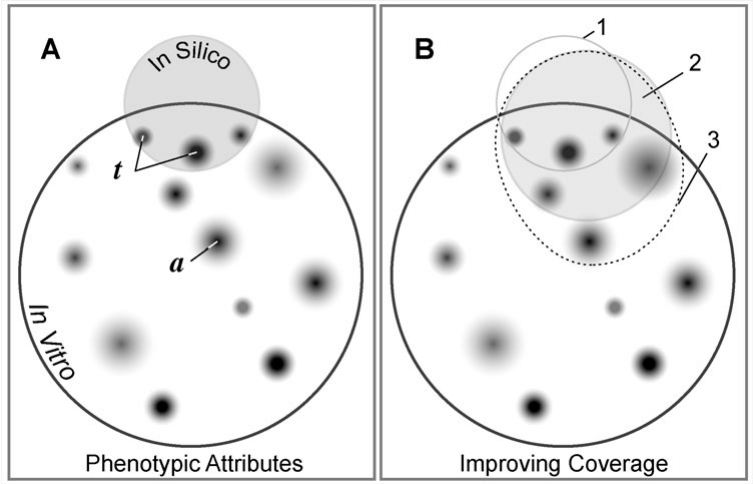
**An illustration of similarities of in silico and in vitro model systems**. An abstract, Venn-like diagram depicts overlapping sets of similar features of in silico and in vitro models. The larger circle is a set of observable, measurable, phenotypic attributes of the experimental in vitro flow chamber system. Attention is focused on selected aspects of the system, e.g., leukocyte interactions with the flow chamber surface. (A) *a*: each small shaded domain represents experimental measures of a specific in vitro characteristic or property such as distance rolled. The degree of shading illustrates different levels of experimental and measurement uncertainty. *t*: these are members of the set of *targeted *in vitro attributes that the in silico model is expected mimic. The shaded circle contains the much smaller set of observable, measurable attributes of a foundational in silico analogue, such as the ISWBC. (B) The sketch illustrates the systematic, sequential extension of the foundational analogue's attributes to improve model-referent phenotype overlap. Circle #1: The set of attributes targeted by the ISWBC in A (the foundational analogue). Circle #2 (shaded): The targeted attributes (represented by circle #1) have been expanded to include two new attributes; however, ISWBC #1 fails to generate behaviors similar to the two newly targeted attributes, resulting in its invalidation. A copy of the ISWBC is iteratively refined (without losing or breaking the original behaviors) by adding new detail only as needed (or by replacing an atomic component with a composite component) until successful coverage of the expanded set of targeted attributes is achieved. Oval #3 (dotted): The analogue represented by circle #2 is to be improved: one new attribute is added to the set of targeted attributes in 2, and the process just described is repeated.

A measure of a particular behavior is a phenotypic attribute. Table 1 lists several targetable attributes. The greater the similarity between the measured behaviors of our in silico white blood cells (ISWBCs) and the in vitro attributes of interest, the more useful that in silico system will become as a research tool and as an expression of the coalesced, relevant leukocyte knowledge. An early goal has therefore been to produce increasing overlap between ISWBC behaviors, properties, and characteristics, and the in vitro properties listed in Table 1. Once that is achieved, we can look forward to simulating behaviors that are more complicated, such as those associated with the above listed diseases, and predicting the consequences of interventions. The phenotypic overlap sought and a method for extending it are illustrated and described in Figure 1. To achieve that overlap we need to enable ISWBCs to mimic an expanding variety of leukocyte attributes. Merging lessons from the literature and related simulation projects [12] with those learned during the early stages of this project, along with identifiable requirements that must be met in order to achieve the insights referred to above, we arrived at ten capabilities that the envisioned ISWBCs should exhibit.

1. *Individual behavior*: the models need to be capable of accurately representing the unique individual behavior patterns typical of leukocytes observed in vitro and in vivo.

2. *Multilevel*^*1*^: it needs to be easy to increase or decrease the number of model levels and alter communications between levels.

3. *Mapping: *there are clear mappings between leukocyte and ISWBC components and their interactions because in silico observables have been designed to be consistent with those of the referent in vitro flow chambers.

4. *Turing test: *when an ISWBC and the simulated flow chamber are each endowed with a specified set of ligands, the measures of ISWBC behaviors during simulations should be, to a domain expert (in a type of Turing test), experimentally indistinguishable from in vitro flow chamber measurements; this requires that an ISWBC and its framework must be suitable for experimentation.

5. *Transparent*: ISWBCs must be transparent. The details of components and their interactions as the simulation progresses need to be visualizable, measurable, and accessible to intervention.

6. *Articulate: *the components articulate. It must be easy to join, disconnect, and replace ISWBC components within and between levels, including the simulated experimental context.

7. *Granular*^*2*^: because of how ISWBCs are componentized, it should be relatively simple to change usage and assumptions, or increase or decrease detail in order to meet the particular needs of an experiment, without requiring significant re-engineering of the in silico system.

8. *Reusable: *an ISWBC (and its components) must be reusable for simulating behaviors in different experimental conditions, in vitro and in vivo, and when different sets of ligands, chemokines, and chemical reagents (e.g., antibodies) are considered.

9. *Embeddable*: ISWBCs must be constructed so that they can function as components of larger, whole organism or tissue models, and eventually represent the full range of trafficking attributes.

10. *Discrete interactions: *to enable the above capabilities, an ISWBC and its framework must use discrete interactions that explicitly show the relation between components.

In this report, we describe significant early progress in building and experimenting with ISWBCs designed to exhibit these capabilities. To achieve them, the ISWBC needed to be distinct from cellular automata (CA) models, including lattice-gas, Ising, and Potts models, in its combination of spatial and non-spatial interactions between components. Some components (agents, as described in Methods) interact via a regular grid as in a CA. However, they also interact directly or through other data structures like lists.

We initially focused on the attributes in Set A of Table 1. We then iteratively revised the ISWBC to mimic the combined attributes in Sets A and B. Each ISWBC was autonomous. Because the preceding capabilities are oriented toward the evaluation of plausible mechanisms, less emphasis is placed on condition-dependent, precise prediction. Behaviors were observed over a range of conditions without modeler intervention. Simulation results were heterogeneous and non-deterministic. Importantly, when simulating populations of leukocytes under different experimental conditions (combinations of substrate molecules), the in silico system generated quantitative population-level data that were similar to data from in vitro experiments. The results have provided an important foundation and framework for future modeling and simulation studies capable of exploring plausible causal links between well-documented, subcellular molecular level events and the variety of system level phenotypic attributes that accurately reflect leukocyte rolling and arrest under a wide range of conditions.

## Results

To avoid confusion hereafter and clearly distinguish in silico components, features, measurements, and events from their in vitro counterparts, such as leukocyte, adhesion, bond, etc., we use SMALL CAPS when referring to the in silico components. Examples are provided in Table 2.

The system described below is the product of iterative refinement of predecessor analogues that focused initially on Set A in Table 1 and later on Sets A and B. Refinement followed the process illustrated in Figure 1B. The in vitro experimental results that we chose to designate as targeted attributes focused on leukocyte ligands PSGL-1, VLA-4 integrin, and CXCR-2 chemokine receptor in addition to their respective substrate ligands: P-selectin, VCAM-1, and GRO-α chemokine. This group represents a minimal set of ligand-binding pairs sufficient for allowing leukocytes to roll, become activated, and firmly adhere in parallel plate flow chambers [3,15-18]. Table 3 lists the leukocyte receptors represented, their corresponding ligands, and the behaviors they support.

**Figure 2:**
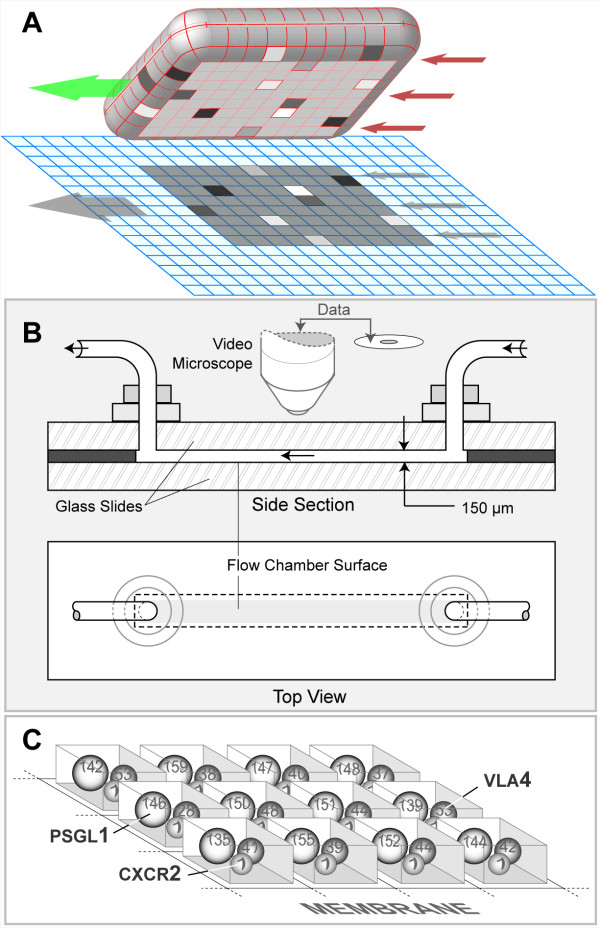
**Sketches of in vitro and in silico experimental system components**. (A) A LEUKOCYTE object is shown pulled away from the simulated flow chamber surface to which it was attached. The left arrow indicates ROLL direction; the three right arrows indicate SHEAR resulting from the simulated flow. The simulated flow chamber surface is discretized into independent units of function called SURFACE UNITS. The LEUKOCYTE'S MEMBRANE is similarly discretized into matching units of function called MEMBRANE UNITS: 600 total (20 × 30). The 8 × 10-shaded region on the SURFACE and on the underside of the LEUKOCYTE identifies the CONTACT ZONE. The UNITS within the CONTACT ZONES that are shaded differently indicate different numbers of BONDS had formed between LIGAND-LIGAND pairs in overlapping UNITS; otherwise, no BONDS formed. ROLLING is the result of a sequence of forward ratchet events. One ratchet event is the result of one row of MEMBRANE UNITS being released at the rear of the CONTACT ZONE along with engagement of a new row of at the front of the CONTACT ZONE. One ratchet event maps to a leukocyte rolling 1 μm (relative to the flow chamber surface). (B) A shop drawing of a typical parallel plate flow chamber used for in vitro studies of leukocyte rolling and adhesion. A video microscope is used to record leukocyte behaviors. (C) MEMBRANE UNITS are illustrated. Each MEMBRANE UNIT is simulated using a software object functioning as a container. In this sketch, nine MEMBRANE UNIT containers are shown. All leukocyte membrane functionality (relevant to these studies) within each UNIT is represented by three objects functioning as agents: PSGL1, VLA4 and CXCR2 (illustrated as spheres). The number of leukocyte ligands being represented by each is typically different from UNIT to UNIT, as illustrated by the numbers on the spheres. That number is specified for each simulation using parameter values from Table 3.

**Figure 3:**
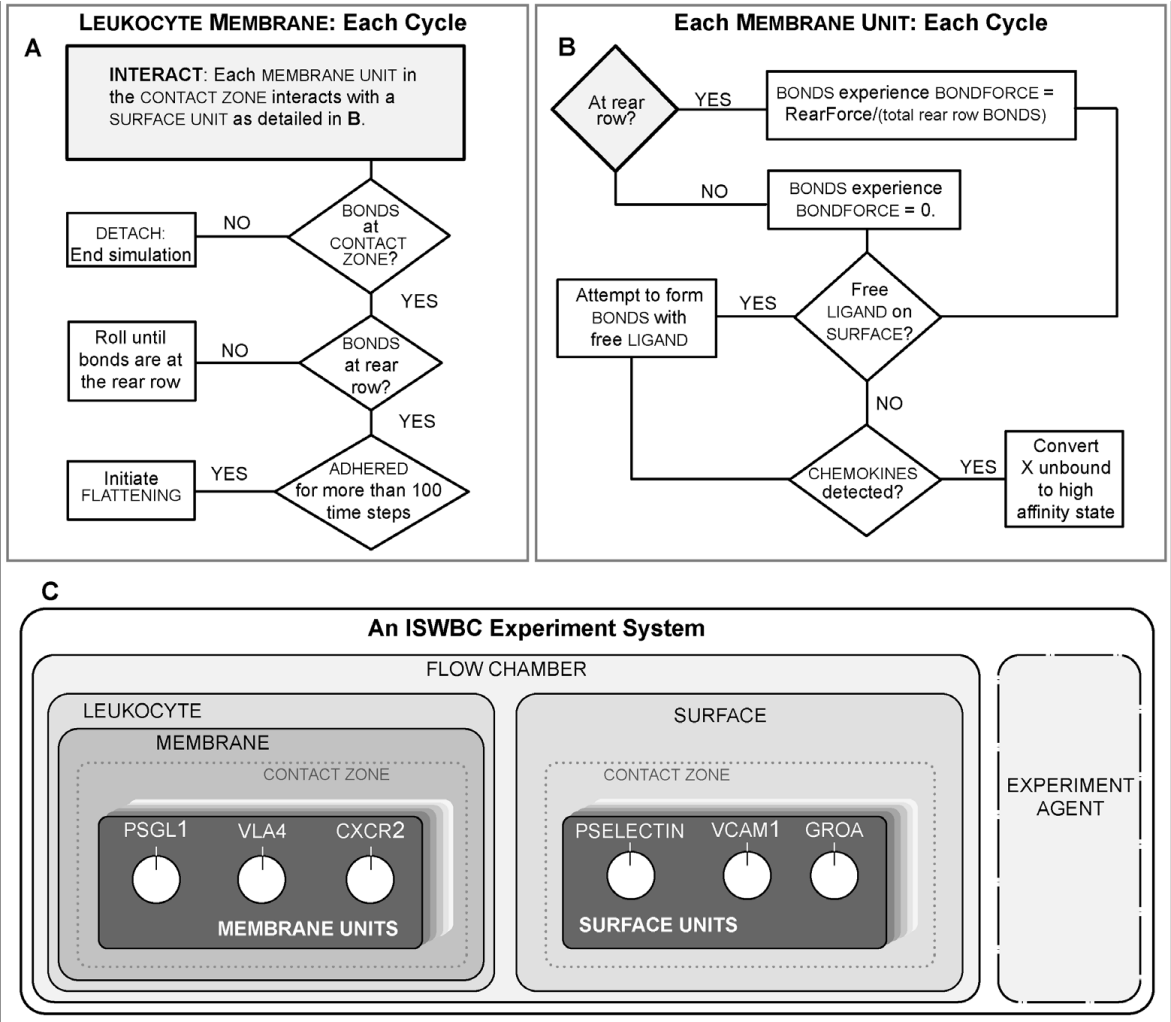
**The decisional process for the LEUKOCYTE MEMBRANE and each MEMBRANE UNIT during a simulation cycle**. (A) The LEUKOCYTE steps through its decisional process only once during a simulation cycle. At the start of the cycle, the MEMBRANE instructs all MEMBRANE UNITS within the CONTACT ZONE to follow the decisional process in B. Once that process is complete, the MEMBRANE completes its process by selecting and following the one applicable action option. (B) A MEMBRANE UNIT is described and illustrated in Figure 2. The state of each depends on the properties of the three LIGAND objects contained within. During each simulation cycle, each MEMBRANE UNIT, selected at random, uses this decisional process to update its status relative to the SURFACE UNIT over which it is positioned. (C) The hierarchical organization of the ISWBC system is illustrated. There are six levels. An Experiment Agent exists within the system, but separate from the FLOW CHAMBER and LEUKOCYTE. It represents a researcher conducting wet-lab experiments: it measures and records events and behaviors during each simulation.

The system illustrated in Figure 2A has been designed to represent an in vitro flow chamber (Figure 2B). The experiments using it are analogous to those performed using an in vitro system (four examples are listed below). The SURFACE of the simulated chamber is "coated" with a uniform density of the LIGANDS of interest. A LIGAND is a component that represents a number of ligands found within a discrete portion (see MEMBRANE UNIT below) of a leukocyte membrane or the flow chamber surface. While in the FLOW CHAMBER, LEUKOCYTES use their LIGANDS to interact and form BONDS with the SUBSTRATE-COATED SURFACE. Each BOND is an in silico analogue of a single ligand-ligand bond. Those interactions are recorded and measured. Under the influence of simulated flow, BONDS at the rear of the CONTACT ZONE experience a simulated cumulative tensile force due to shear; it is controlled by the parameter *RearForce*. We learned from earlier, simpler analogues that a dynamic decisional process of the type diagrammed in Figure 3, was needed at two levels: LEUKOCYTE/MEMBRANE and MEMBRANE UNIT. As described in Methods, the SURFACE, LEUKOCYTE, and MEMBRANE are separate objects. A MEMBRANE UNIT is an analogue of a functional unit of leukocyte membrane within the CONTACT ZONE. There is a corresponding SURFACE UNIT. The number of UNITS is a discretization decision. How fine or course does the discretization (granularity) need to be in order to avoid discretization artifacts and achieve the targeted behaviors? As shown below, a level of discretization is reached at which an acceptable match to targeted behaviors is achieved; further increasing granularity does not improve the quality of the match.

We investigated the ISWBC's ability to represent in vitro measures of rolling and adhesion by comparing simulation results with data from three different flow chamber experiments:

(1) Smith et al. [16] and Park et al. [15] observed human neutrophils rolling on various densities of P-selectin and under varying wall shear rates.

(2) Alon et al. [17] recorded human lymphocyte-rolling trajectories on VCAM-1 in the presence of increasing wall shear rates at fixed time intervals.

(3) Monocyte rolling and adhesion on P-selectin and/or VCAM-1 in the presence or absence of GRO-α chemokine was studied by Smith et al. [3].

### Simulating neutrophil rolling on P-selectin

The in vitro data suggests that leukocyte rolling is mediated by small numbers of receptor-ligand bonds formed within the contact zone. Mathematical model estimates of this number range between two and twenty [19,20]. Single bond events are believed to cause rolling behaviors similar to what one might expect of a stochastic process. There are two easily recognized manifestations: jerky stop-and-go patterns and highly fluctuating rolling velocities [2]. Given the importance of single bond events, each ISWBC BOND maps to a single ligand-ligand bond. However, a LIGAND can map to more than one ligand molecule.

Two observations considered together, pause times and forward movement during rolling, required that the upper limit for the mapping of simulation cycle to in vitro time be about 1 simulation cycle = 0.1 seconds. Pause time measurements, the first targeted attribute, were made over a small range of shear values as reported by Smith et al. [16] for human neutrophils rolling on a P-selectin-coated surface. Using high-speed videomicroscopy with a smallest resolvable step size of 0.5 μm, average pause times were in the range of 0.1 to 0.16 seconds for shear stress values of 0.5 to 2.0 dyn/cm^2^. Higher wall shear stress values caused shorter average pause times.

**Figure 4:**
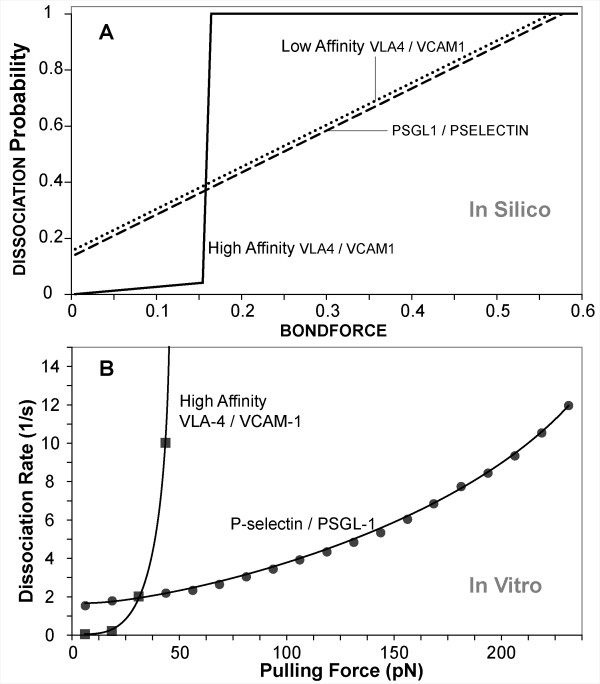
**FORCE dependence on BOND DISSOCIATION probability and force dependence on bond dissociation rates**. (A) Shown is the relationship between *bondforce *and probability of BOND DISSOCIATION for each of the three LIGAND pairs within the CONTACT ZONE. The effects of shear on the ligand-ligand bonds that form at the rear of the leukocyte are simulated using *bondforce*. BONDS within the rear row of the CONTACT ZONE experience a *bondforce *that is calculated by dividing the *RearForce*, a unitless parameter representing the effects of shear, by the total number of BONDS within the rear row. During a simulation cycle, each MEMBRANE UNIT in the rear row uses the current value of *bondforce *and the graphed relationship to calculate a probability that each BOND will be broken during that cycle. All BONDS elsewhere within the CONTACT ZONE experience a *bondforce *value of 0. UNSTRESSED (*bondforce *value of 0) DISSOCIATION probabilities for PSGL1/PSELECTIN, LOW-AFFINITY VLA4/VCAM1, and HIGH-AFFINITY VLA4/VCAM1 were chosen to be 0.14, 0.16, and 0.0035, respectively. (B) The in vitro force dependence of dissociation rates for P-selectin/PSGL-1 bonds (as reported in [15]) and the high affinity VLA-4/VCAM-1 bonds (as reported in [29]) are plotted for comparison to the analogue relationships in A. The plotted values were taken from the fitted in vitro data: see Methods for details. The relationships in A are analogues of these experimentally determined relationships and are not meant to either match or fit that data. The dissociation rates of the PSGL-1/P-selectin bonds as a function of force were determined by experiments using PSGL-1-coated microbeads rolling on a P-selectin substrate in a parallel plate flow chamber [15]. The dissociation rates for the VLA-4/VCAM-1 complex, described in Methods, were calculated from data obtained using single-molecule dynamic force spectroscopy [29]. The force dependence of dissociation rates for low affinity VLA-4/VCAM-1 data was not reported. We assumed that it is similar to PSGL-1/P-selectin relationship in A.

**Figure 5:**
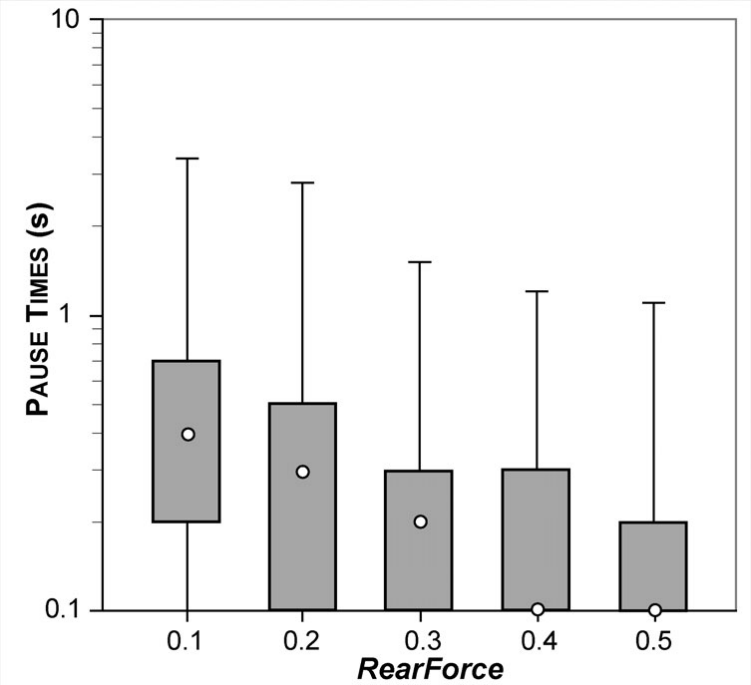
**Boxplot of measured PAUSE TIMES for LEUKOCYTES ROLLING on PSELECTIN at various *RearForce *values**. At each *RearForce *value, average PAUSE TIME was recorded from 60 simulations that had at least 10 INTERACTIONS (pauses). In vitro, higher values of wall shear stress lead to shorter pause times. This data shows that higher *RearForce *values also lead to shorter LEUKOCYTE pause times. White circles: median pause time value. Box: lower and upper quartiles. Whiskers: minimum and maximum pause time values.

We defined LEUKOCYTE ROLLING as a sequence of at least 10 simulation cycles during which a LEUKOCYTE remained non-stationary on the SURFACE. During ROLLING, ISWBCs used the relationship between LIGAND disassociation probability and *bondforce *graphed in Figure 4A. *Bondforce *is an in silico analogue of the force experienced by each BOND within the contact zone. When parameterized according to Table 4, using the values listed in Table 5 (part I-A), the simulation's smallest forward movement (ratcheting forward by one MEMBRANE UNIT) maps to 1 μm, twice that of the videomicroscope used by Smith et al. To be acceptably similar, given the difference in time resolution, the magnitude of average simulated pauses needed to be within 1 to 2 times the in vitro values of 0.1 to 0.16 seconds, or between 0.1 and 0.32. That was achieved when we specified 1 simulation cycle = 0.1 seconds in vitro. Figure 5 shows that at *RearForce *values of 0.2 through 0.5, the median PAUSE TIMES fell within that range. We judged these results to be acceptable. At a *RearForce *value of 0.1, the median PAUSE TIME appeared to deviate slightly from this range. Future studies using a finer time resolution may yield results even closer to the in vitro data, should that be needed.

**Figure 6:**
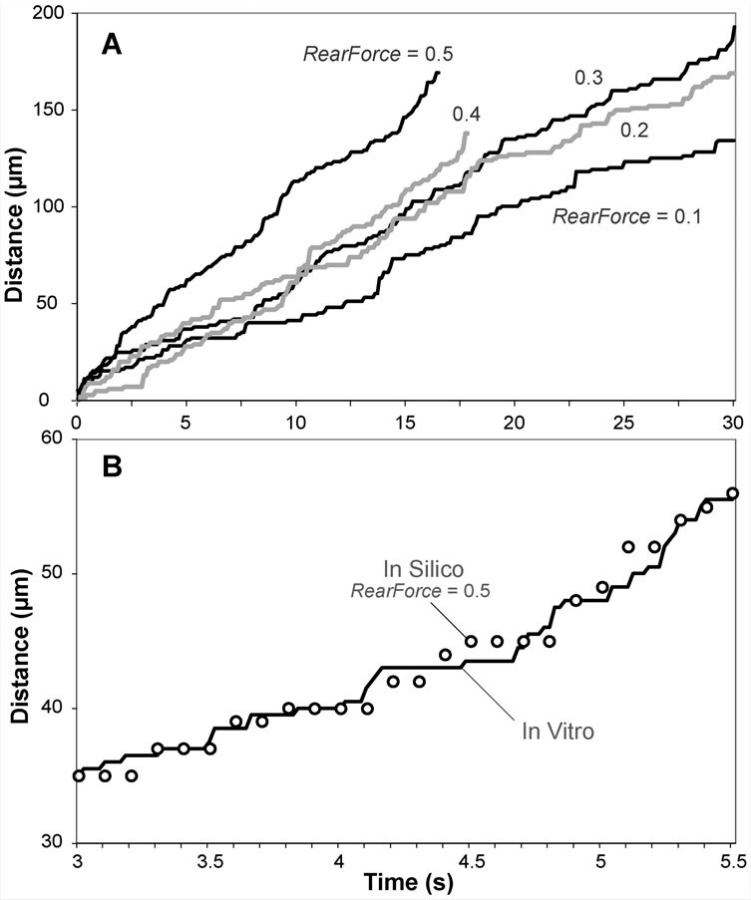
**LEUKOCYTES ROLLING on PSELECTIN exhibit the characteristic jerky stop-and-go pattern of leukocyte rolling in vitro**. (A) Examples are graphed for distance-time plots for a single ROLLING LEUKOCYTE studied in each of the indicated five *RearForce *conditions using the ENVIRONMENT parameter values in Table 5 (part I-A). (B) Solid line: values of a single leukocyte trajectory as reported in [16]. Open circles: an example LEUKOCYTE trajectory from simulations that used the ENVIRONMENT parameter values in Table 5 (part I-B).

Simulated distance-time measures for each *RearForce *condition exhibited the characteristic jerky stop-and-go movement (Figure 6A). Smith et al. reported trajectories of human neutrophils rolling on P-selectin at a density of 25 sites/μm^2 ^while under a wall shear stress of 2.0 dyn/cm^2^. When using the ENVIRONMENT parameter values from Table 5 (part I-B), simulations produced LEUKOCYTE trajectories that were experimentally similar to in vitro data (Figure 6B).

**Figure 7:**
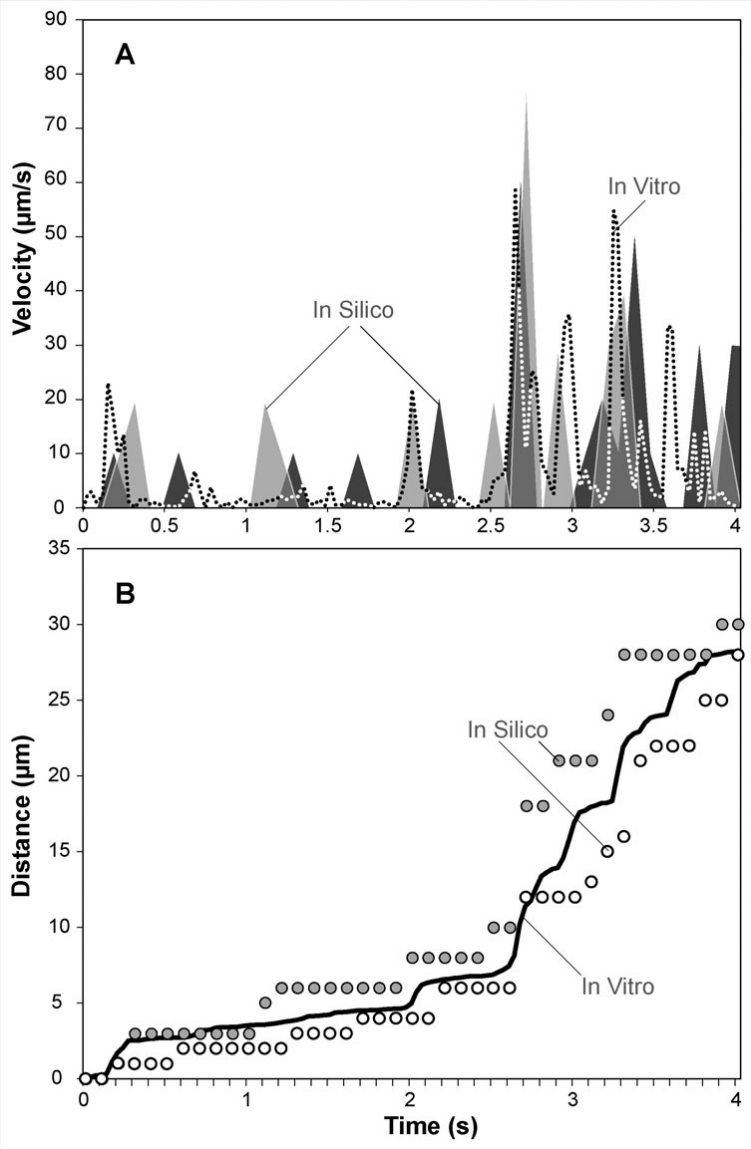
**Comparison of in silico and in vitro instantaneous velocity data**. (A) Dotted trace: a leukocyte rolling on P-selectin in vitro as reported in [15]. Gray, shaded traces: two simulations of a LEUKOCYTE ROLLING on PSELECTIN using ENVIRONMENT parameter values from Table 5 (part I-C). Both traces show fluctuating rolling velocities similar to the dotted trace. (B) Distance-time plots from the experiments in A; solid line: in vitro leukocyte (calculated from the reported instantaneous velocity data); circles: the two in silico LEUKOCYTES from A.

Simulated velocity-time measures also showed that LEUKOCYTES exhibited fluctuating velocities similar to those reported in the literature. Park et al. [15] observed human neutrophils rolling on P-selectin at a density of 9 sites/μm^2 ^under a wall shear rate of 0.5 dyn/cm^2^. When we ran simulations using ENVIRONMENT parameter values from Table 5 (part I-C), we observed similar behavior: the range of both LEUKOCYTE ROLLING velocity values and distance values plotted in Figure 7 are similar to those reported in [15]. The results clearly exhibit the essential jerky stop-and-go movement and highly variable ROLLING velocities that are characteristic of leukocytes rolling.

To further evaluate and validate LEUKOCYTE ROLLING, we determined the average ROLLING velocity, the average number of BONDS within the contact area, and the average number of BONDS at the rear of the LEUKOCYTE. Table 6 shows results. Average ROLLING velocities fell within ranges that have been reported for rolling on P-selectin. Consistent with in vitro experimental data, average ROLLING velocity increased with increasing *RearForce *values. The average number of BONDS within the contact area and the average number at the rear of the LEUKOCYTES under all three *RearForce *conditions were consistent with the estimated numbers stated above.

### Experiments that simulate lymphocyte rolling on VCAM-1

VLA-4 is unique among the integrins, as it has been shown to support leukocyte tethering and rolling. In flow chambers coated with VCAM-1, a ligand for VLA-4, leukocytes roll even in the absence of selectins or chemokines [3,17]. Competitive binding experiments determined that native state VLA-4 has affinity constant values similar to those of the selectins [21].

**Figure 8:**
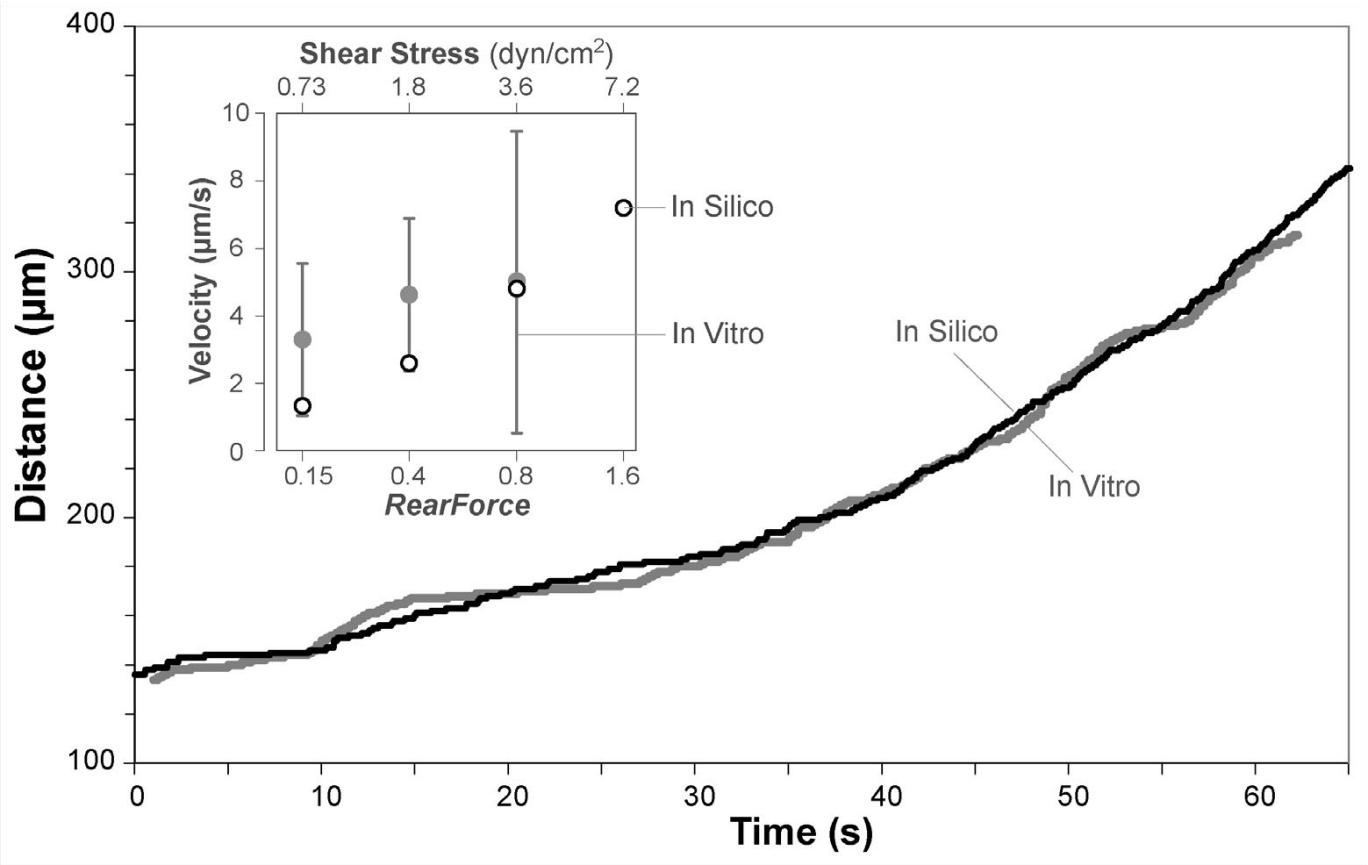
**Simulating experimental condition 2: rolling on VCAM-1 with shear increased at fixed intervals**. Alon et al. [17] observed T-lymphocytes rolling on VCAM-1 in the absence of chemokine under increasing wall shear stress; wall shear stress was increased at fixed intervals causing increased leukocyte rolling velocities. Black line: a leukocyte trajectory reported in [17]. Gray line: an example LEUKOCYTE trajectory when using ENVIRONMENT parameter values from Table 5 (part II). Insert: in vitro measures of leukocyte velocity for different values of shear (upper axis); the standard deviations (vertical bars) were conservatively estimated using the standard errors reported in [17]. Circles: average ROLLING velocities of LEUKOCYTES (n = 60) for different *RearForce *values (lower axis) fall within the in vitro ranges. The original published distance-time plots begin at ~140 microns.

The goal of the second set of simulation experiments was to show that ISWBC ROLLING behaviors are similar to those of leukocytes rolling on VCAM-1 in the absence of chemokine. Alon et al. observed human T-lymphocytes rolling on different densities of purified VCAM-1 in flow chambers [17] under increasing shear stress. Shear was increased at fixed intervals resulting in increased leukocyte rolling velocities. Using parameter values from Table 4 and Table 5 (part II), simulations generated LEUKOCYTE ROLLING trajectories that appeared indistinguishable to those reported by Alon et al (Figure 8). Average ROLLING VELOCITIES were calculated for each *RearForce *value. They were within the range of in vitro rolling velocities at each reported shear stress value (Figure 8, Insert).

### Simulating monocyte rolling, activation, and adhesion on P-selectin and/or VCAM-1 in the presence or absence of GRO-α chemokine

**Figure 9:**
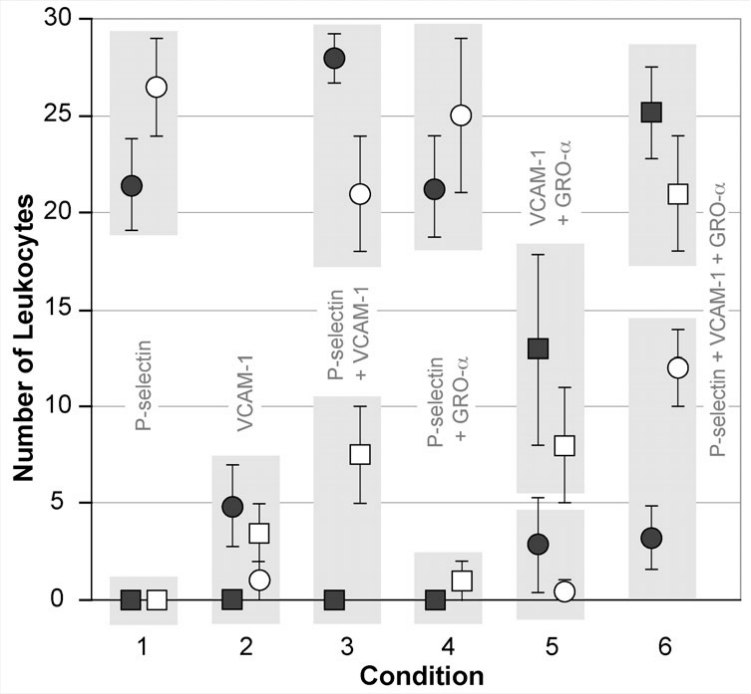
**Comparison of in vitro and in silico results for six different experimental conditions**. The in vitro conditions (from [3]): the flow chamber surface was coated with P-selectin and/or VCAM-1 with or without immobilized GRO-α chemokine. The number of leukocytes that rolled and adhered within each of five fields of view were recorded for a 30-second observation interval. In vitro: white circles: average number of leukocytes that rolled; white squares: average number of leukocytes that adhered; error bars: ± 1 SD. The data are clustered and plotted for each of six conditions, as labeled. By using the parameter values in Table 5 (part III), the in silico experiments mimicked the in vitro experimental conditions and also the results: the results are averages from 20 sets containing 30 LEUKOCYTES each; each simulation ran for 300 simulation cycles (equivalent to about 30 seconds). In silico: dark circles: average LEUKOCYTES that ROLLED; dark squares: average LEUKOCYTES that ADHERED; error bars: ± 1 SD. Each light gray box contains the two sets of observations (in vitro and in silico) that should be compared.

Smith et al. [3] used a flow chamber to observe human monocytes rolling and adhering on P-selectin and/or VCAM-1 with or without immobilized GRO-α chemokine: six different conditions were studied. During perfusion of leukocytes through the chamber, 30-second recordings of five fields of view were taken along the chamber length. The average number of rolling and arrested cells was determined. Cells that remained immobile for at least 20 seconds were considered arrested. Using that same combination of in silico substrate analogues, the ISWBC parameter space, constrained by the already established parameter values in Table 5 (parts I and II), was searched empirically for parameter sets that would provide acceptable matches for all six experimental conditions. The LEUKOCYTE parameters from Table 4 and the ENVIRONMENT parameters from Table 5 (part III) is such a set; it produced the results in Figure 9: the data are averages from 20 sets of experiments containing 30 LEUKOCYTES each, with the duration of each run being 300 simulation cycles (equivalent to about 30 seconds). The number of ROLLING and adhering LEUKOCYTES for each batch were counted and averaged. LEUKOCYTES that remained stationary on the SURFACE for at least 200 simulation cycles (about 20 seconds) were classified as ADHERED. Figure 9 shows that for all ligand combinations simulated, the data matched that from in vitro fairly well. There was even a similar significant increase in the number of ADHERENT LEUKOCYTES on PSELECTIN and VCAM1 due to the presence of GROA CHEMOKINE, as was observed in vitro.

There are some notable discrepancies between the in silico and in vitro data that are most likely a consequence of an ISWBC being an abstract simplification of the more complex reality. For example, in the absence of GRO-α chemokine, Smith et al. observed a small fraction of cells that were able to adhere when rolling on VCAM-1 alone or when VCAM-1 was co-immobilized with P-selectin. This small fraction of adhering cells may be attributable to the pre-activation of monocytes during cell isolation procedures. We do not include any pre-activation effects, and consequently do not observe any ADHERING LEUKOCYTES in the absence of GROA CHEMOKINE. By affecting adhesion, the pre-activation of monocytes may additionally affect the number of rolling leukocytes on VCAM-1 substrate. We had such considerations in mind when we adjusted parameter values, and so did not seek the best possible match for any one condition. Instead, we focused on obtaining behavioral similarities under different conditions. By adjusting parameter values to bring in silico and in vitro observations closer together for one condition, we typically amplified discrepancies for other conditions. If narrowing these discrepancies is deemed among the important issues to be addressed next, then our approach would be to include that specification within the targeted attribute list in Set C of Table 1. To achieve that goal, additional ISWBC detail would likely be required. Literature guided, exploratory simulations would be needed to identify candidate details. Fortunately, because ISWBC design and construction are being guided by the ten Capabilities listed in the Introduction, such exploratory simulations are relatively easy to pursue.

**Figure 10:**
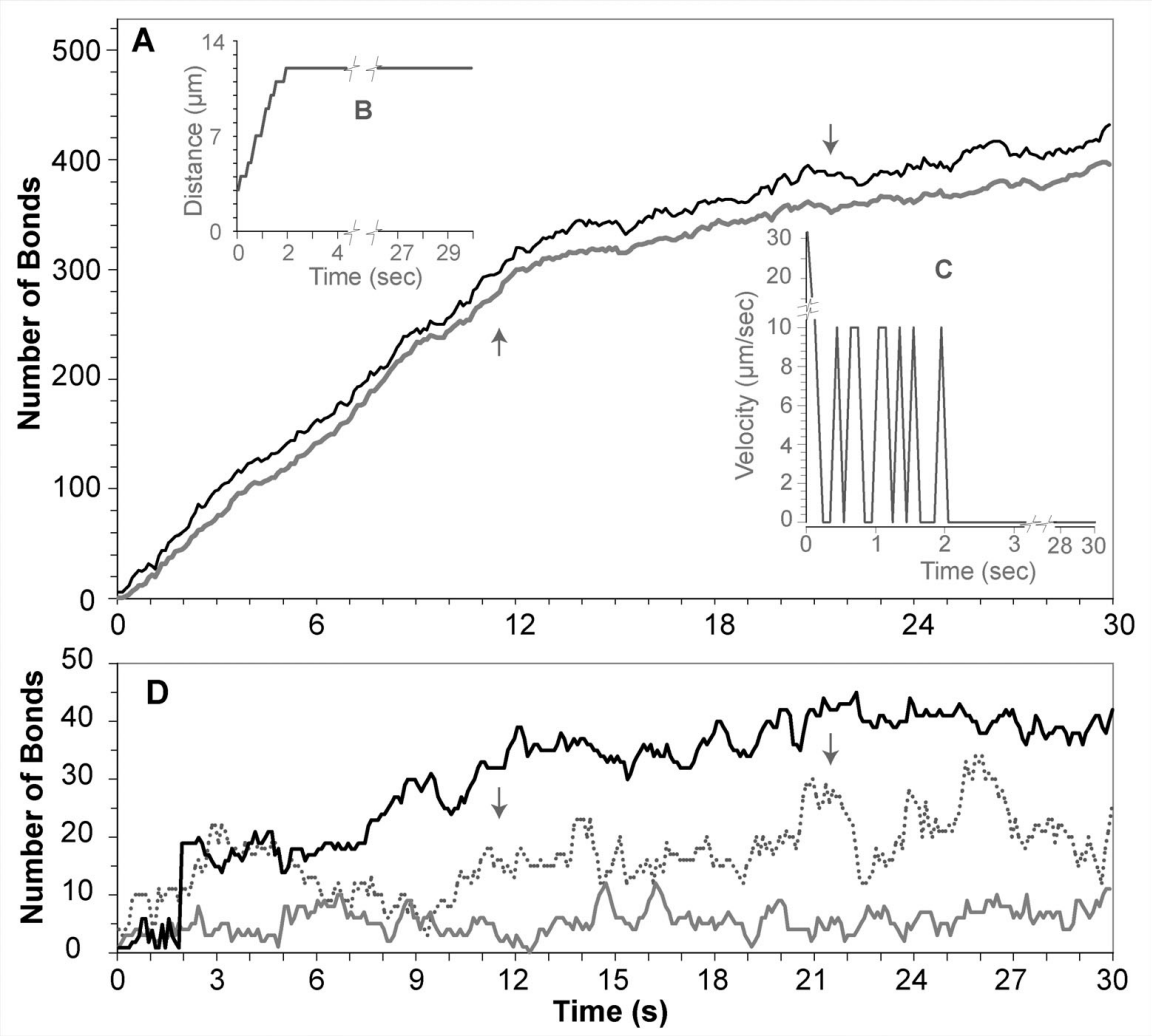
**Measurements from a LEUKOCYTE that ROLLED and ADHERED to PSELECTIN and VCAM1 after activation by GROA CHEMOKINE**. Smith et al. [3] observed that when the flow chamber surface is coated with P-selectin and VCAM-1 co-immobilized with GRO-α chemokines, monocyte arrest occurred within a few seconds. The data graphed is from an in silico experiment that simulated that protocol and is for an individual LEUKOCYTE that transitioned from ROLLING to FIRM ADHESION (Table 5, part III). (A) Dark line: total number of BONDS formed within the CONTACT ZONE between LEUKOCYTE MEMBRANE and SURFACE. Light gray line: number of high affinity VLA4-VCAM1 BONDS formed: these results show that ADHESION is mediated primarily by the high affinity VLA4-VCAM1 BONDS. (B) DISTANCE-TIME plot and (C) VELOCITY-TIME plot: they show that the LEUKOCYTE rolled for less than a few simulated seconds before firmly adhering to the SURFACE, consistent with leukocyte adhesions observed in vitro. (D) For the same experiment, the number of low affinity VLA4-VCAM1 BONDS and PSELECTIN-PSGL1 BONDS are small and so are plotted here at a smaller scale. Dotted trace: number of low affinity VLA4-VCAM1 BONDS. Lower solid gray trace: number of BONDS formed between PSELECTIN and PSGL1. The black trace is a separate plot of the total number of BONDS formed at each time in the rear row of this CONTACT ZONE; these data show that for the ISWBC, the LOW AFFINITY VLA4/VCAM1 AND PSELECTIN/PSGL1 BONDS play only a minor role in supporting ADHESION. Arrows indicate when a LEUKOCYTE MEMBRANE SPREADING event occurred.

Smith et al. also tracked individual monocytes under flow to obtain rolling and arrest profiles [3]. They observed that with P-selectin and VCAM-1 co-immobilized with GRO-α chemokines, monocyte arrest occurred within a few seconds. We simulated their protocol: we tracked individual LEUKOCYTES that were able to transition from ROLLING to FIRM ADHESION. We also observed that FIRM ADHESION on PSELECTIN and VCAM1 in the presence of GROA CHEMOKINE was primarily mediated by high affinity VLA4 and that it occurred within SECONDS. Figure 10 shows an example LEUKOCYTE that is able to ADHERE after ROLLING.

## Discussions and conclusion

### Setting the stage

A long-term biological goal for this project is to develop scientifically useful, validated simulations of leukocyte recruitment under physiological and pathophysiological conditions. The envisioned LEUKOCYTES, functioning within an analogue of the wet-lab context, will have many behaviors, properties, and characteristics that mimic those of referent in vitro and in vivo systems. Stated differently, there will be similarities between leukocyte phenotype and the "phenotype" of simulated leukocytes, as illustrated in Figure 1. The expectation is that increasing phenotype similarity will require, and can be achieved in part through, similarities in design plan and in generative mechanisms. Once they are achieved, the model will have become a scientifically useful analogue.

Our approach to designing and building the envisioned analogues is motivated by aspect-oriented software development and the middle-out approach first suggested by Brenner [22] and later detailed by Noble [23] for building models of complex biological systems. We adapted and merged these ideas in arriving at our working definition of middle-out modeling. First, specify the biological features under study (e.g., cell rolling and attachment to a surface). Each feature becomes an aspect of a software device – an archetype model. That archetype is iteratively transformed into a functioning analogue of the referent in vitro biological system. It is then validated and improved iteratively.

Following that approach, our first task was to pick a set of properties, p_1_, p_2_, ... p_j _(within a hyperspace of mechanistic properties) – around leukocyte rolling and adhesion in vitro. We specified Set A, Table 1. We next asked what software device could we implement to realize p_1_? How can we realize p_2_, etc.? We then asked, how can we realize p_1_- p_j_, all at the same time, using the same software device? Getting answers to those questions required exploratory modeling and simulation, occasionally using different modeling and simulation support packages. The in silico properties and the device we created to realize p_1_-p_j _formed a foundational analogue, the above archetype model, from which we intended to expand outward (up, down, and even sideways) to establish a reductive hierarchy of components that could be used to identify important systems biology principles.

### Creating an analogue phenotype

The ISWBC is an abstract model of a leukocyte, and when placed in an appropriate, simulated environment, it is capable of mimicking a set of targeted characteristics. The ISWBC exists within a system that is a model of an in vitro flow chamber system (cells, media, the device in Figure 2B, atmosphere, etc.). The ISWBC is a multilevel composite model. The components are independent models of their referent leukocyte or environment counterpart. For example, the in silico component representing the MEMBRANE UNITS in a portion of the trailing edge of a ROLLING LEUKOCYTE is itself a model of the corresponding leukocyte feature.

The envisioned relationship between measured LEUKOCYTE behaviors and corresponding in vitro measures is illustrated in Figure 1. Pictured are overlapping (but not intersecting) sets. One set (Figure 1, large circle) contains the results of experiments that measured specific phenotypic attributes, properties, or characteristics of leukocytes in vitro (e.g., all three sets in Table 1). The smaller sets contain the results of simulation experiments that measure corresponding in silico phenotypic attributes, properties, or characteristics (such as Set A, Table 1). Our motivating hypothesis has been that, when attention is focused on the same features, and the measured behaviors of the two sets are similar, then there may also be useful similarities in the generative mechanisms of the two systems. Those similarities can be explored by iterative testing and refinement of the analogue coupled with related wet-lab experiments. An example: distance traveled by a LEUKOCYTE in silico (or a leukocyte in vitro) under various simulated (or actual) shear stresses. We cannot expect these measurements to overlap completely or even have the same units. Observations on behavior similarity can be used simultaneously to eliminate invalid model features and identify gaps in our knowledge of the experimental in vitro system.

The first step in transitioning from an archetype to an improved LEUKOCYTE was to thoroughly document the in silico properties and characteristics of the former. By doing so, we improved insight into its strengths and weaknesses. The next step was to obtain increasing overlap with measures of in vitro attributes. That was done systematically by iteratively revising together the hypothesis and the analogue by following the five steps below. An example hypothesis: the current analogue can acceptably simulate the first two attributes [p_1_, p_2_,] in Set A, Table 1. Figures 7 and 8 are examples of successful simulations. We begin the next iterative cycle with step 1.

1) Select an additional in vitro property or characteristic that is related to those already in the target set, and for which wet-lab experimental observations are available, such as the third property in Table 1. Identify the additional property as p_3_.

2) Add p_3 _to the targeted set, yielding an expanded set [p_1_, p_2_, p_3_], as illustrated by 2 in Figure 1B.

3) Determine if addition of the new attribute invalidates the current analogue, and if so, why. If not, repeat step 2.

4) Revise the model iteratively, possibly by adding mechanistic detail, until the measured phenotypic attributes of the revised analogue are sufficiently similar to [p_1_, p_2_, p_3_].

5) The next iterative cycle repeats steps 1–4 with p_4_, etc.

Our approach to achieving an envisioned ISWBC was strongly influenced and constrained by the ten capabilities listed in the Introduction. We reasoned that to achieve our long-term goal it would be essential for future ISWBC descendents to exhibit those capabilities.

### Design

The conceptual model of the referent system, which is fundamental to the design of the in silico model, is as follows. Functionalities within the leukocyte membrane are grouped into a large number of similarly capable modular, functional units. Leukocyte rolling in vitro involves a balance between the hydrodynamic force exerted by fluid flow and the resistant forces resulting in part from transient bonds being formed between the leukocyte and the substrate. Shear force coupled with random events causes bonds to break at the rear of the contact zone. Bonds formed within the contact zone are by nature transient and will dissociate even in the absence of a tensile force. Only a few bonds need to be present at any time in order to maintain a rolling interaction. In order to maintain that interaction, broken bonds must be replaced by new ones formed elsewhere within the contact zone.

The transition from rolling to adhesion is mediated by the integrin receptors that, when induced into a high affinity state, are capable of forming effective ligand interactions that are sufficiently stable to withstand the hydrodynamic fluid force. However, they exist natively in a low affinity state and can only aid in supporting rolling interactions. There are many hypothesized mechanisms of integrin activation. We explored one of the simplest: a chemokine receptor, upon binding to its chemokine ligand, induces a local VLA-4 integrin molecule into a high affinity state. The resulting high affinity integrins form stronger and longer-lasting bonds with VCAM-1. Diffusion and clustering of VLA-4 integrins is also hypothesized to play a role in firm adhesion. However, to keep things relatively simple at this early stage, we have not simulated those events.

### Achievements

The ISWBC components have been verified, plugged together, and operated in ways that may represent (at a relatively high level) mechanisms and processes that influence leukocyte rolling and adhesion. The approach has allowed us to represent the spatial and discrete event phenomena that are thought to occur during leukocyte rolling, activation, and adhesion. It has allowed us to represent apparently stochastic elements within the system that may play a vital role in determining leukocyte behavior *in vivo *and *in vitro*. Our earliest archetype had a CONTACT ZONE comprised of 2 × 3 MEMBRANE UNITS (48 for the total MEMBRANE of the LEUKOCYTE). With it, we failed to even approach the first two targeted attributes in Set A, Table 1; we were unable to simulate the stochastic fine structure present in the in vitro data because the discretization was too coarse. It is common to observe such discretization artifacts during simulation development when the granularity is too coarse. To better achieve the targeted behaviors, we decreased the relative MEMBRANE UNIT size (decreased granularity) to represent the CONTACT ZONE by 3 × 6 units and then in steps to the current size of 8 × 10 units (600 UNITS for the total MEMBRANE). With each increase in granularity, simulated results more closely mimicked the in vitro data, as judged by inspection. We also explored even finer grained CONTACT ZONES, up to 20 × 30, without further gains in similarity. Taken together, these results suggest that membrane functionality within and adjacent to the contact zone is distributed into many (from our results, about 80) equally capable, somewhat autonomous units.

Though the ISWBCs are relatively simple, we have demonstrated that they can generate the targeted attributes under three different experimental conditions. Traditional models typically focus on just one condition. ISWBCs mimic the dynamics of leukocytes rolling separately on a P-selectin or VCAM-1 substrate. In addition, they mimic the transition from rolling to adhesion on P-selectin and VCAM-1 in the presence of GRO-α chemokine. Importantly, when simulating populations of leukocytes under different experimental conditions (combinations of substrate molecules), the system generates quantitative population-level data that are similar to data from in vitro experiments.

One hypothesis of leukocyte activation suggests that leukocytes roll along the vessel wall progressively engaging with chemokine signals to reach a global activation threshold. Once achieved, fully activated integrins can then support firm adhesion. However, there is growing support for an alternative hypothesis: leukocyte adhesion induced by immobilized chemokines occurs via local and spatially restricted signaling to nearby integrins. Our results support the latter hypothesis. In an elegant study, Shamri et al. [24] showed that leukocyte engagement with chemokine locally and reversibly induced the β_2 _integrin, LFA-1, into an intermediate affinity state that was essential for interaction with ICAM-1 under flow conditions. Concurrently, engagement with ICAM-1 induced the signaling events needed to fully activate local LFA-1 to the high affinity state needed to support firm adhesion. Because of the modeling approach used (providing the ten capabilities, etc.), it will be relatively easy for future descendents of the ISWBCs to represent such fine-grained, cooperative behaviors and spatially restricted events.

### Appraisal of the model assumptions

We have simulated human leukocytes of slightly different sizes (neutrophils, lymphocytes, monocytes), however we kept the CONTACT ZONE the same for all simulations. It has been observed that leukocytes of greater size have a greater area of surface contact, and thus can form more bonds that are adhesive. However, biomechanical analysis shows that with increasing leukocyte size there is an increase in the amount of force and torque experienced. This higher magnitude is believed to translate to higher forces experienced by bonds. Cozen-Roberts [25] presented an analysis that was verified in vitro, which predicted that the disruptive force increases faster than the pro-adhesive effect of increased contact area. Rather than changing the dimensions of the contact area when simulating experiments with different leukocyte types and sizes, we fixed that dimension and compensated by using greater *RearForce *values to represent the same shear force when simulating larger leukocytes (see Table 5).

The conceptual model above is widely accepted for neutrophils. However, it is not entirely known how well it applies to other leukocyte types, such as lymphocytes and monocytes. Varying receptor types and densities found on the different leukocyte types may be a factor contributing to differing mechanisms of adhesion. For example, lymphocytes and monocytes express basal levels of VLA-4 integrins sufficient to support rolling [17]. In contrast, it has been shown that transmigration across endothelium or exposure to chemotactic stimuli is necessary to induce expression of VLA-4 integrins on human neutrophils [26]. Therefore, rolling and adhesion through the VLA-4 integrin may require additional or different mechanisms for neutrophils than for lymphocytes and monocytes. Our objective during this project was not to construct a comprehensive model of rolling, activation, and adhesion for all leukocyte types. However, when simulating monocyte rolling and adhesion, we assumed that monocytes and neutrophils share similar rolling characteristics on P-selectin. Likewise, we assumed that the characteristics of monocyte rolling on VCAM-1 are similar to those for lymphocytes. The only difference was presumed to be the densities of each receptor type found on each leukocyte type. Parameter values representing the densities of the VLA-4 integrins for lymphocytes in experimental condition 2 and monocytes in experimental condition 3 were different to reflect the different VLA-4 density values reported in the literature. Values for PSGL-1 sites/human monocyte were not found in the literature and were assumed to be similar to values reported for human neutrophils.

### Comparison to other leukocyte rolling and adhesion models

The discrete-time models used in the Adhesive Dynamics simulations by Hammer and co-workers are the most developed models of leukocyte rolling and adhesion to date [9-11]. Leukocytes in their models are idealized as solid spheres decorated with rod-like microvilli containing receptors at their tips. Their simulations have allowed them to explore the molecular properties of adhesion molecules, such as reaction rates and bond elasticity, and how these properties may relate to macroscopic behavior such as rolling and adhesion [9,11].

In their simulations, the position of the cell is determined at each time step from the net force and torque on the cell using a hydrodynamic mobility function for a sphere near a plane wall in a viscous fluid. The net force and torque acting on the cell from bonds, fluid shear, steric repulsion, and gravity are calculated. When calculating the forces on the sphere due to fluid shear, their model uses the Goldman equation [27]: the amount of force a sphere experiences from an applied shear force is calculated by assuming that the sphere is solid. At each time step in the Adhesive Dynamics simulation, positions of bonds on the spherical particle are tracked enabling the authors to calculate the forces that each bond experiences.

It was not our intention to discover ISWBC parameterizations that would yield simulation results that tightly fit specific sets of experimental data. Our approach has been focused on simulating a targeted set of system-level properties (Figure 1). Therefore, we did not, for example, attempt an explicit mapping between the shear force and the amount of force experienced by bonds at the rear of the leukocyte. Leukocytes are notoriously deformable and therefore we elected to avoid a mapping such as the Goldman equation. In addition, the mapping from the force on the cell to the force on the rear bonds can be quite complex, as PSGL-1 and VLA-4 are both ligands that are concentrated at the tips of stretchy and heterogeneous microvilli. It should be noted that shear stress for selectin tethers at the rear of neutrophils have been estimated previously using the Goldman equation to be 124 ± 26 pN per dyn/cm^2 ^[28].

To account for the effect of an applied force on the kinetics of bond dissociation, Hammer and co-workers have employed both the Bell model and Dembo Hookean Spring model (see [2]). The Bell Model predicts bond dissociation rate as a function of applied force, whereas the Dembo model treats bonds as Hookean springs and relates dissociation rate to the length of the stretched bond. Use of a similar fine-grained representation of bond dissociation may have yielded more precise simulation results, but that level of resolution and precision was not needed to meet the simulation objectives in Table 1. Rather, we used a simple model to relate the probability of bond dissociation with bond force (Figure 4A); it was motivated by in vitro experiments by Park et al. [15], and Zhang, et al. [29]. Because the time resolution within the ISWBC is low (a tenth of a second), using the linear approximations in Figure 4A proved reasonable: at small bond force values, we assumed a linear relationship between the probability of bond dissociation and bond force. At larger bond force values, we reasoned that the rates of bond dissociation for ligands are too fast to be distinguished on our time-scale. Consequently, BONDS experiencing larger forces are represented as having undergone a DISSOCIATION event during a simulation cycle with a probability of 1.

Many molecular level details are believed to impact effective bond formation and breakage within small portions of a leukocyte membrane and the surface. Examples include contact irregularities, local dynamics, including ligand relocation within the membrane, force history of bond loading, and bond compliance. All are aggregated and controlled in the ISWBC by an event probability. When explanation of system level behaviors requires a more detailed representation, one or more of these factors can be specifically represented, without compromising the function of other ISWBC components. The probability parameters will remain, but their values and explanation will have changed.

In the most recent version of their model, Hammer and co-workers represent the p38 mitogen-activated protein kinase (MAPK) global integrin activation mechanism leading to leukocyte arrest induced by E-selectin engagement with its ligand PSGL-1 [30]. A simple modular Hill function is used to represent the overall MAPK cascade leading to integrin activation. At the end of each time step, the total number of E-selectin-PSGL-1 bonds are counted and used as an input to this function. We have chosen to explore an alternative integrin activation mechanism in which detection of chemokines activates local integrins. Future embodiments of this modeling effort may include the E-selectin-dependent signaling pathway.

Recent experiments have shown that leukocyte rolling on substrate-coated, flow chamber surfaces is significantly slower and smoother than that of microspheres coated with ligands [15,31]. It has been hypothesized that the deformability of leukocytes can influence rolling by increasing the contact area. In vivo, leukocytes rolling at blood wall shear rates of 800 s^-1 ^elongate in the direction of flow to 140% of their estimated undeformed diameter and have a 3.6-fold increased contact area with the endothelium than at blood wall shear rates of 50 s^-1 ^[32]. These experiments have influenced other modelers to explore the mechanical and rheological properties that influence leukocyte morphology and deformation. The 2-D elastic ring model [33] and the 2-D compound drop [34] model offer an account for the deformability of leukocytes during rolling. However, because of the way receptor-ligand interactions are treated deterministically, these models do not produce the characteristic jerky stop-and-go behavior of rolling leukocytes.

Recently, Jadhav et al. created a 3-D model that provides a representation of leukocyte deformability: it can mimic the stochastic nature of receptor-ligand interactions [35]. The model uses a mathematical formulation called the Immersed Boundary Method to simulate the measured motion of a leukocyte by representing it as an elastic capsule in a linear shear field. They use a Monte Carlo method for simulating receptor-ligand interactions, enabling them to simulate the jerky stop-and-go movement of rolling leukocytes. Their model explored the effects of membrane stiffness on cell deformation and leukocyte rolling. It is believed that the contact area between the surface and the leukocyte membrane would increase for leukocytes under increasing shear rates. By introducing deformation in their model, they are able to observe this change in contact area. Our model currently ignores this phenomenon: we kept the contact area fixed for all simulations. Future ISWBC descendents can, when needed, allow each CELL to vary its own contact area.

Considerable knowledge has been gleaned from these different models of leukocyte rolling and adhesion. They have provided insight into how molecular parameters of adhesion molecules or cell deformability may influence leukocyte rolling and adhesion. The knowledge gained reinforces the merit of investigating complex phenomena using different modeling methods. Our approach is quite different and is aimed at studying the interactions of the components and possible mechanisms that may give rise to emergent leukocyte- and systems-level phenotypes.

### Computational models of cell motility

Beyond the primarily mechanical models considered above, what other modeling methods might one consider to achieve the goal presented above under Setting the Stage? Large-Q Potts models are based on extensions of cellular automaton (CA) models [36], including lattice-gas CA, with features adapted from the Ising model. They have been used, for example, to study the cell sorting of embryonic cells [37], and extended to simulate a variety of different biological phenomena [38]. A further extension using a layer for partial differential equations (describing cAMP dynamics) enabled elegant simulations of the complex cell movements occurring during culmination of the morphogenesis of the slime mould *Dictyostelium discoideum *[39]. Importantly, the simulated cells were represented as a group of connected automata making the basic model scale subcellular. Important details of these methods are collectively reviewed by Alber et. al [40].

Such extended Potts models have been the preferred approach for describing cell motility at a subcellular level, including aspects of cell shape, surface chemistry, and internal structure. Nevertheless, Meyer-Hermann and Maini found it necessary to develop an alternative, agent-oriented model architecture to interpret recent the two-photon imaging data of lymphocyte movement within lymph nodes [41]; the jerky and individualistic nature of the imaging data is similar to that of leukocyte rolling. They refer to their system as the hyphasma (the Greek word for tissue) model.

The hyphasma LYMPHOCYTE is a connected collection of subunit objects arranged on a 2D square grid centered on a virtual barycenter. Each one has a polarity vector, a list of its constituent subunits, internal velocity states, and a CELL volume that determines the number of subunits. During movement, the barycenter is shifted in the direction of the polarity vector and border subunits are randomly moved to lattice points near the newly established barycenter. The simulated results exhibit Capability 4 (*Turing Test*) and support the hypothesis that lymphocyte movement within secondary lymphoid tissue may not be a consequence of chemotaxis or haptotaxis, but a simple random walk. It seems likely that the hyphasma model could be used to simulate data like that in Figures 5, 6, 7, 8. However, extending the model so that it delivers Capability 3 (*Mapping*) seems problematic. Relative to the abstract nature of the subunits, the authors state: "a force balance equation for the cell subunits is not necessarily a correct description of active processes of deformation and reshaping of cells. Thus, the interpretation of cell subunit velocities in terms of forces has to be considered as an approximation to more complex internal processes within the cell." Simulating data like that in Figures 9 and 10 is beyond hyphasma's current capabilities, but then the model was not developed with the intent that it be adaptable and extensible to represent such data (Capabilities 5 and 8). As stated in Introduction, when developing the ISWBC we had in mind objectives beyond simply simulating the in vitro data presented. We sought and presented a method for assembling individual components (that map to identifiable biological counterparts) according to a design, *and *then showed that the constructed ISWBC can exhibit behaviors that match those observed.

### Future directions

The ISWBC described here is an important first step towards our long-term goal of building in silico devices that can simulate leukocyte behaviors in a variety of in vitro experimental systems even in the face of considerable uncertainty. They are useful objects for experimentation, as are the referent in vitro models. The current ISWBC is capable of mimicking only a few (Table 1) of a long list of desired phenotypic attributes of leukocytes observed in vivo and in vitro. The expectation is that ISWBCs can be iteratively refined to become increasingly realistic in terms of both components and behaviors. Because we strove to deliver the ten capabilities listed in the Introduction, any of the above, abstract, low resolution components can be replaced with validated, more realistic, higher resolution composite objects that map to more detailed biological counterparts, without having to reengineer the whole system, and without having to compromise already validated features and behaviors.

## Methods

### An in silico device

One of the best ways to understand a complex system is to build a device, an analogue, which exhibits some of the same behaviors. We cannot build scientifically useful cell-like devices from inanimate organic components, but because of advances in software engineering, we can build cell-like devices in software. The in silico system described below is such a device. It is a discrete event, discrete space, and discrete time analogue of the entire in vitro parallel plate flow chamber system sketched in Figure 2B. We used the synthetic modeling method [42-45]. Object-oriented software components were designed, verified, and then plugged together in ways that were intended to represent the modules, components, mechanisms, and processes that are believed to influence leukocyte adhesion (or lack thereof) and rolling in vitro. We have tried to keep the gap between in silico mechanism and measured phenomena as small as practicable. In so doing, we avoided getting too complicated too soon. We have used the Recursive Porous Agent Simulation Toolkit (RePAST) as our modeling and simulation framework. It is a java-based software toolkit developed at the University of Chicago for creating and exercising agent-based models. The libraries provided are used to create, run, display, and collect data. From within the RePAST framework, the system described below can operate in ways that represent the hypothesized mechanisms and processes at the level of detail and resolution needed simulate the targeted attributes (Sets A and B) in Table 1.

### In silico system components

**Figure 11:**
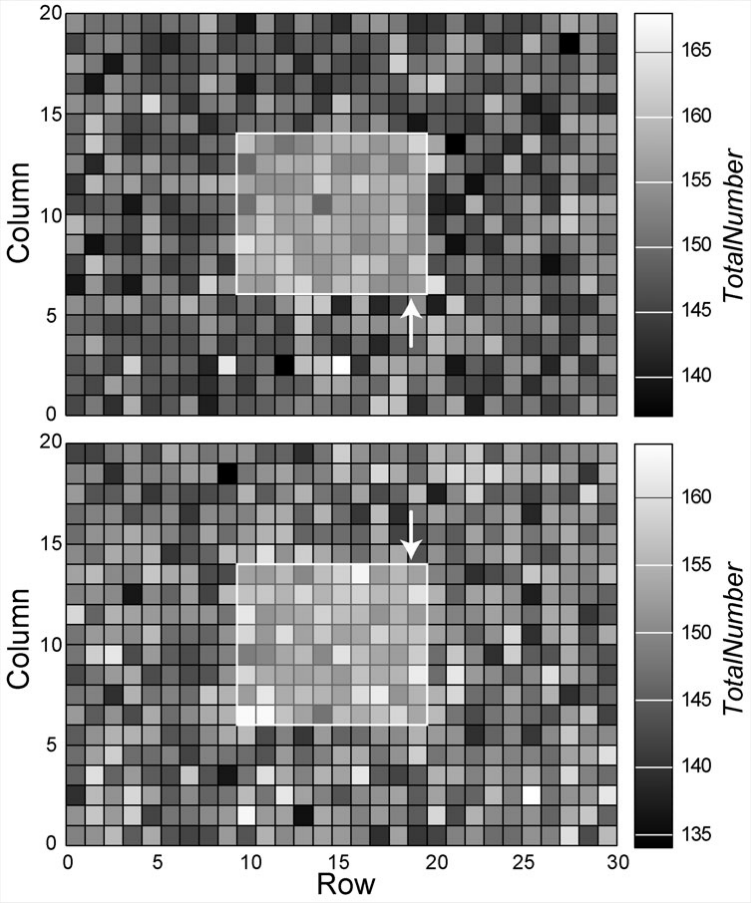
**Contour plots show the number of PSGL-1 molecules represented by a MEMBRANE UNIT**. The number of PSGL-1 represented are shown for two different LEUKOCYTE MEMBRANES. Each of the 600 MEMBRANE UNITS within each MEMBRANE contains one PSGL1 agent (see Figure 2C). Each PSGL1 *TotalNumber *parameter value (indicated by the scale on the right) specifies the number of PSGL-1 molecules represented in a specified MEMBRANE UNIT. When averaged over the entire MEMBRANE, a typical PSGL1 represents 150 ± 5 PSGL-1 (see Table 3). The 8 × 10 region outlined in white is the CONTACT ZONE. Arrows specify the row of MEMBRANE UNITS (at the "rear" of the CONTACT ZONE) that experiences *RearForce*, when the LEUKOCYTE is moving from right to left.

The reliance on grids (Figures 2A and 11) in the details that follow, where different locations can have different properties, is reminiscent of CA. However, the ISWBC is not a CA, nor is it similar to extensions of CA, such as hybrid CA, lattice-gas CA, and Potts models. A CA [36] consists of an infinite, regular grid of cells, each in one of a finite number of states. The grid can be in any finite number of dimensions. Time is discrete. The state of a cell at time t is a function of the states of a finite number of cells (its neighborhood) at time t-1. The neighborhood does not change. Every cell has the same rule for updating, based on the values in this neighborhood. Each time the rules are applied to the whole grid a new generation is created.

A CA is not a grid with several automata in it. The whole collection, cells, logic, and data, constitute a single automaton. This differs from the ISWBC, in which objects reside and interact, and in which each agent can act autonomously (both within and between spaces) and, e.g., set their own schedules and determine the other agents with whom they will interact. A CA takes place on a regular grid and has a fixed mediation space that is globally defined and does not change. Extended CA, such as a hybrid CA, might have several grids (or irregular grids, like a Voronoi grid) that may come into play at different times.

Rather than attempt several extensions of the CA concept, we draw on concepts used in object-orientation software design and are built into agent-based modeling methods. Object-orientation design (irrelevant of programming) is the most natural technology in which to render an in-depth analogous modeling relation. To achieve all ten capabilities we need a multi-scale, hierarchical, discrete-event, discrete-time, object-oriented, agent-based modeling method that relies on message passing and partially ordered sets of events for object interaction. By using the minimal formalism of partially ordered sets, such a model can maximize its expressibility and minimize artifacts that may be caused by formalisms that are more restrictive, such as CA and its extensions.

Each LEUKOCYTE is represented by an object (Figure 2A). Models of leukocyte adhesion often assume that the molecular interactions within the area of contact between leukocytes and the substrate-coated surface are the only relevant interactions during rolling and adhesion. We do the same. We assume that, because of the required, locally fast reaction times, all required intracellular processes occur quickly, or are localized close to the membrane. We assume that during rolling and adhesion, intracellular processes work together to enable behaviors. Consequently, for the attributes targeted, we treat intracellular processes as being constant and MEMBRANE UNIT processes (defined below) as being autonomous.

We refer to that portion of a leukocyte membrane in contact with the surface as the contact zone. Leukocyte membrane functionality within the contact zone is heterogeneously distributed. Some receptor types are confined to microvilli tips, whereas others are found in the spaces between. We discretized the leukocyte membrane by subdividing the contact zone into arbitrary units of functionality (hereafter, membrane unit). All of the functionality within a membrane unit (signaling to and from the cell interior, endocytosis, secretion, etc.) is aggregated, and represented by the behavior of a MEMBRANE UNIT. That behavior is an emergent property of the objects (or agents) contained within (see Figure 2C). The granularity of that subdivision need only be fine enough to represent the targeted behaviors (Table 1). Because of having specified the *articulate *and *granular *capabilities (5 and 6: Introduction), it was easy to increase or decrease subdivision granularity.

Because the targeted attributes involve only the contact zone, only events that specifically influence those behaviors are represented. Other aspects of membrane dynamics and molecular biology are not ignored. They are simply not among the system aspects on which we have focused in this project. Any of them can be brought into focus when needed by adding one or more new components to each MEMBRANE UNIT, and that can be done without having to completely reengineer the ISWBC. There are currently only three objects within each MEMBRANE UNIT: one to represent the functionality of each of the three different leukocyte membrane ligands: PSGL-1 ligand, VLA-4 integrin, and CXCR-2 chemokine receptor. Each of these objects is an agent^3^.

For convenience and simplicity, we represent the contact zone using an arrangement of MEMBRANE UNITS in a uniform grid (Figure 2A), and refer to it as the CONTACT ZONE. A MEMBRANE UNIT within the CONTACT ZONE maps to an arbitrary portion of membrane that is in contact with the surface, and may include the tethers that are known to form between rolling leukocytes and surfaces [46]. Leukocytes have stretchy microvilli and are highly irregular in shape [46-48]. Consequently, the precise area of membrane-surface contact during rolling is uncertain. We reflect that uncertainty by specifying that MEMBRANE UNITS are changeable, and by not specifying a mapping between a MEMBRANE UNIT and a precise amount of membrane area. However, the mapping between MEMBRANE UNIT and distance rolled is important: that is discussed below under Behavior. The balance of the LEUKOCYTE'S surface outside the CONTACT ZONE is assumed a reservoir of MEMBRANE UNITS. For programming convenience, we represented that reservoir as an extension of the CONTACT ZONE, as illustrated in Figure 2A, that has grid dimensions 5–10 times that of the size of CONTACT ZONE. Hereafter, we refer to the entire grid as the MEMBRANE. To accommodate MEMBRANE mobility relative to the SURFACE, the MEMBRANE at the software level is a 2D toroidal lattice.

The number of MEMBRANE UNITS within the CONTACT ZONE can be easily increased or decreased. The observed ratio of length to width of rolling rat leukocytes in vivo is approximately 1.25-1.5 [47] over a range of shear values. We assumed that rat and human leukocytes deform similarly, and therefore represented the contact zone using a rectangular grid with width-to-length dimensions having a similar ratio. All simulations in this report represent the CONTACT ZONE using an 8 × 10 arrangement of MEMBRANE UNITS. That resolution (level of granularity) was selected after experimenting with coarser and finer representations. Larger granularities failed to exhibit the targeted behaviors^4 ^(within a factor of two or better). Smaller granularities failed to improve simulation results. For two reasons, we did not optimize CONTACT ZONE size. First, we have not yet developed a means of determining precisely which of two similar behaving but differently parameterized simulations is the closer match over a range of in vitro measures (such as Figures 5, 6, 7, 8). Second, LEUKOCYTE behavior also depends on the nature and specification of the components within (discussed below).

A portion of the bottom flow chamber surface is represented by the SURFACE SPACE (hereafter simply SURFACE). SURFACE functionality was also subdivided. In each simulation, its granularity matches that of the MEMBRANE, as illustrated in Figure 2A: one unit of SURFACE corresponds to about 1 μm^2^. That grid is also a 2D toroidal lattice. As with the MEMBRANE, each unit of SURFACE function is represented by a container object, called a SURFACE UNIT, one per grid space. Contained within each SURFACE UNIT are agents representing the substrate molecules (P-selectin, VCAM-1, and GRO-α chemokine) for the three leukocyte membrane ligands listed in Tables 2 and 3.

Agents contained within MEMBRANE UNITS and SURFACE UNITS are of the class type LIGAND. A LIGAND agent represents a group of binding molecules of the same type that may be found within a portion of the membrane or flow chamber surface. For example, a PSGL1 represents several PSGL-1 adhesion molecules. The number represented is illustrated by the numbers assigned to the objects within each MEMBRANE UNIT in Figure 2C. The actual number represented is determined by the parameter *TotalNumber*. At the beginning of each simulation, its value is uniquely determined according to the values of *DensityMean *and *DensitySTDev*. Figure 11 shows the *TotalNumber *values of the PSGL1 for two MEMBRANES used during a simulation. The targeted behaviors were achieved using random assignment of values to each PSGL1 and VLA4 agent. Particular patterns of ligands within the contact zone, such as regions of high and low PSGL-1 densities can also be implemented when required by targeted behaviors.

Table 3 shows LIGANDS sub-classed as ADHESION MOLECULES, CHEMOKINE RECEPTORS, and CHEMOKINES, corresponding to adhesion molecules, chemokine receptors, and chemokines, respectively. In addition, there is a sub-class of ADHESION MOLECULES called INTEGRINS. INTEGRINS are different from the other LIGANDS in that they have two sets of parameters, one represents a low, and another represents a high affinity state. All INTEGRINS are initially in the low affinity state. Events within the CONTACT ZONE (see below) determine if, and when, an INTEGRIN is converted to the high affinity state.

For each ligand type, on either the chamber surface or leukocyte membrane, there can be a corresponding LIGAND agent within the container object at each SURFACE and MEMBRANE grid location. We have set a maximum value of one LIGAND of each type at all locations. Table 2 lists each of the ligands in the model, the LIGAND objects that represent them, their LIGAND class type, and their location within the ISWBC.

### Behaviors

During each simulation, the CONTACT ZONE portion of the MEMBRANE is positioned "above" the SURFACE so that LIGANDS in overlapping MEMBRANE and SURFACE UNITS can "see" and interact with each other (Figure 2A) and form BONDS.

When the bonds on tethers and other points of contact are broken at the rear of a rolling leukocyte under the influence of shear, the leukocyte ratchets forward some distance (until sufficient new bonds are formed, as discussed below). The ISWBC works analogously. If there are no BONDS within the rear row of the CONTACT ZONE, the LEUKOCYTE ratchets forward (north) until the rear row contains at least one BOND during the simulation cycle. Ratcheting involves removing a row of MEMBRANE UNITS from the rear of the CONTACT ZONE while a new row is placed at the front. Thus, the location of the CONTACT ZONE on the MEMBRANE and its position "over" the SURFACE changes whereas CONTACT ZONE dimensions are kept constant (Fig 3A). During a single simulation cycle, a ratchet movement can be repeated up to nine times (for a LEUKOCYTE with a CONTACT ZONE length of 10 MEMBRANE UNITS). Currently, only forward (north) movement is permitted. However, we can enable east or west rolling-like movements when that is needed. We have also implemented SURFACE boundary conditions in order to reduce the number of simulated SURFACE UNITS. A LEUKOCYTE ROLLING off any edge will continue ROLLING at the opposite edge. This feature has reduced computational time and simplified visualization.

We assume that each ratchet movement maps, on average, to a leukocyte having moved 1 μm. If the data were available to support doing so, the mapping between each forward ratchet and relative distance moved could be drawn from a distribution having a mean of ~1 μm and some specified variance. By so doing, we may represent some of the real uncertainty associated with in vitro measures. However, the data are not yet available. For simplicity, we fixed the mapping to be 1 μm. If the mapping between a simulation cycle and in vitro time is changed, then this distance mapping too must change.

Components have been designed to facilitate changing CONTACT ZONE dimensions dynamically during a simulation. It is believed that leukocyte deformation supports firm adhesion by increasing the zone of contact, thereby allowing formation of more adhesive bonds. If a LEUKOCYTE remains ADHERENT for at least 100 simulation cycles, an extra row is added at the front and an extra column is added along one side (selected randomly) of the CONTACT ZONE allowing additional BONDS to form. This process mimics spreading and helps stabilize ADHESION.

During each simulation cycle, each LIGAND within the CONTACT ZONE has one opportunity to form and break BONDS. However, only BONDS at the rear row of the CONTACT ZONE experience the effects of the SHEAR FORCE (described below). Only when all BONDS at the rear have been BROKEN can a LEUKOCYTE ROLL. If at any time (during any simulation cycle), there are no intact BONDS within the CONTACT ZONE, the LEUKOCYTE removes itself from the SURFACE and the simulation ends, mimicking a leukocyte detaching and being swept away by the fluid flowing through the chamber. Figure 3 outlines the programmed decisional processes that determine the flow of events within each simulation cycle.

### Forming and Breaking BONDS Between ADHESION MOLECULES

When a LEUKOCYTE LIGAND encounters its binding partner on an overlapped SURFACE UNIT, it first determines the theoretical maximum number of BONDS that can be formed (defined below). A Monte Carlo algorithm is then used to determine the actual number of BONDS that form. Each simulated adhesion molecule type uses the values of three parameters to determine BOND-formation with its binding partner: 1) *TotalNumber *specifies the number of receptors. The smaller of the two *TotalNumber *parameters is used during calculations of the theoretical maximum. 2) *BondNumber *specifies the number of BONDS that already have been formed between the LIGAND pair. The theoretical maximum number of BONDS is simply the difference between these two values. 3) *Pon *is the probability of bond formation for each binding pair. *Pon *was fixed at 0.001, 0.001, and 0.005 for PSGL-PSELECTIN, low affinity VLA4-VCAM1, and high affinity VLA4-VCAM1 BONDS, respectively.

For each potential BOND, *Pon *is compared to a pseudo-random number between 0 and 1 to determine if the BOND is actualized. *Pon *is fixed for the duration of a simulation. For the INTEGRINS that represent ligands with high affinity states, this process is repeated separately using parameter values corresponding to the higher affinity state.

The effect of shear on the rear of a leukocyte is represented by the variable *RearForce*. BONDS experience a *bondforce *that is calculated each simulation cycle by dividing the *RearForce *by the total number of BONDS in the rear row of the CONTACT ZONE. Bonds within the rest of the CONTACT ZONE experience no *bondforce*. Drawing from the in vitro data discussed below, we have assumed the simple linear relationship between *bondforce *and the probability of BOND dissociation shown in Figure 4A. It is calculated as (probability of dissociation) = b_0 _+ (*bondforce*) × b_1_, where b_1 _and b_0 _are the slope and intercept, respectively, of the line segment associated with a specific *bondforce*. Each type of simulated adhesion molecule pair uses a unique set of b_0 _and b_1 _values obtained from Figure 4A. Each relationship is intended to be an analogue of the force dependence of dissociation rates for P-selectin/PSGL-1 bonds reported by Park et al. [15], and those of high affinity VLA-4/VCAM-1 bonds reported by Zhang et al. [29] (Figure 4B). The *bondforce *relationships in Figure 4A are not intended to match or precisely fit in vitro data. Doing so would require that we specify the relationship between wet-lab measures of bond force and our in silico parameter *bondforce*. There is neither need nor reason to do so at this early stage of analogue development.

Park et al. calculated PSGL-1/P-selectin dissociation rates as a function of force experienced by the bond by observing PSGL-1 covered microbeads rolling on P-selectin substrate in a parallel plate flow chamber. For the range of dissociation rate constants relevant to this report (*Koff *values < 10/s), the in vitro data, as shown in Figure 4B, is close to linear (the values in Figure 4B were calculated from the reported, best fit values [15]). The unstressed dissociation constant, *K*^0^*off*, for PSGL-1/P-selectin bonds were calculated to be 1.6/s.

Zhang et al. used single-molecule dynamic force spectroscopy to investigate the strength of the VLA-4/VCAM-1 complex. The experimental conditions were not the same as in vitro. Nevertheless, the relative behavior of the VLA-4/VCAM-1 complex is expected to be similar to that in the flow chamber. The dissociation of the VLA-4/VCAM-1 complex was determined to involve overcoming two activation energy barriers. Under pulling forces < ~50 pN, dissociation rates were governed principally by the properties of the outer activation energy barrier. The *K*^0^*off *values for the outer energy barriers for the low- and high-affinity complexes were 1.4/s and 0.0035/s, respectively. The high affinity data in Figure 4B were converted from the reported semi-log plots [29]. The force dependence of dissociation rate for low affinity VLA-4/VCAM-1 was not reported. We assumed that it is similar to PSGL-1/P-selectin, and represented it so in Figure 4A.

### Activation

A LEUKOCYTE that is interacting with the SURFACE uses CHEMOKINE RECEPTORS to detect any CHEMOKINE object that may be present. As a simplification (and because each simulation cycle is long compared to chemokine binding and dissociation), we specified *Pon *and *Poff *values of 1.0 for CHEMOKINE RECEPTORS such that each detected CHEMOKINE can BIND and DISSASOCIATE (i.e., the events are registered as having been detected) within the same simulation cycle. Consequently, CHEMOKINES can be detected by the same RECEPTOR during different simulation cycles. For each CHEMOKINE detected, a message is sent to the INTEGRIN within that MEMBRANE UNIT to decrement the low affinity *TotalNumber *parameter and increment the high affinity *TotalNumber *parameter. If low affinity *TotalNumber *reaches 0, then the activation mechanism for that INTEGRIN is turned off.

A maximum of approximately 10% of β-2 integrins on a leukocyte membrane become induced into high affinity states when exposed to chemokines [49]. We have assumed that the VLA-4 integrin has similar properties. When the sum of the *TotalNumber *high affinity state parameters from all INTEGRINS within the MEMBRANE exceeds 12.5% of the sum of the *TotalNumber *high and low affinity values from all the INTEGRINS, the activation mechanism is turned off for all INTEGRINS. The above representation of the chemokine dynamics is a simple, abstract metaphor for a complex phenomenon. Future ISWBC descendants will include representations of chemokine binding and signaling dynamics that are more realistic.

### Software

The ISWBC software and support documentation is available at [58].

## Appendix

### Footnotes

^1 ^Replacing an object within the model with a collection of subunit objects that still give rise to the original object behavior is an example of increasing model level. See [13] for a discussion of hierarchical, modular modeling methods, and [14] for a review of the related topic of multi-scale models.

^2 ^When we increase or decrease the number of grid points in the CONTACT ZONE, we are changing granularity. That has no impact on the number of model levels.

^3 ^Technically, an agent is a software object (either atomic or composite) with the ability to interact with its environment and schedule its own actions.

^4 ^An example of an unacceptable behavior was too frequent detachment. If we make the properties and behaviors of the components within a MEMBRANE UNIT more complicated, we can get similar behaviors from a contact area comprised of fewer units. However, a goal is to keep component objects as simple as possible.

## Abbreviations

ISWBC: in silico white blood cell; CA: cellular automata (automaton)

## Authors' contributions

In consultation with CAH, JT conceived the model, designed experiments, implemented the simulation, and then analyzed and opined simulation results. KL contributed data that was simulated by earlier versions of the model. CAH and JT wrote the paper with input from KL. All authors read and approved the final manuscript.

## Figures and Tables

**Table 1:** Targetable phenotypic attributes of leukocytes in vitro: an abridged list

**Phenotypic Attribute**	**Figure/Table**	**Reference**
*Set A: initially targeted attributes*		
Jerky stop and go movement	Figures 5, 6	[16]
Highly fluctuating rolling velocities	Figure 7A	[15]
Larger rolling velocities observed at higher shear rates	Figures 6A, 8	[50]
Smaller rolling velocities at higher ligand substrate densities	Not Shown	[50]

*Set B: additional attributes added to targeted set*		
ROLLING^a ^velocities on PSELECTIN^a ^match reported values	Table 6	[50]
Small number of bonds within the contact zone, e.g., within 2–20	Table 6	[19], [20]
Distance-time and velocity-time data for ROLLING^a ^on ^a^PSELECTIN/VCAM1 are indistinguishable from reported data	Figures 6, 7, 8	[15], [16]
Chemokines induce adhesion within seconds	Figure 10	[3]
Leukocyte spreading during firm adhesion	Figure 10	[51]

*Set C: future, targetable attributes*		
Ligand-Receptor mobility, valency (clustering), and trafficking	N/A	[5], [6], [52]
Change in contact surface area with increasing shear force	N/A	[25], [53]
Shear threshold effect for selectins (minimum shear required for rolling)	N/A	[50]
Effect of inhibitors to signaling molecules on cell arrest (pertussis toxin (PTx)-sensitive G proteins, phosphoinositide 3-kinase, p38 mitogen-activated protein kinase)	N/A	[3]
Intracellular calcium mobilization	N/A	[3]

During early development of the ISWBCs attention focused primarily on Set A. Once satisfactory simulations began to be achieved, the targeted attributes were expanded to those in Set B. Final validations focused on the combined set.

^a ^We use SMALL CAPS when referring to the in silico components (see Table 3), features, measurements, and events.

**Table 2:** In vitro and in silico ligand counterparts, their class types, and their locations within the ISWBC

**Ligand Molecule**	**In Silico Model Component**	**Object Class**	**Location**
PSGL-1	PSGL1	ADHESION MOLECULE	MEMBRANE
P-selectin	PSELECTIN	ADHESION MOLECULE	SURFACE
VLA-4	VLA4	INTEGRIN	MEMBRANE
VCAM-1	VCAM1	ADHESION MOLECULE	SURFACE
CXCR-2	CXCR2	CHEMOKINE RECEPTOR	MEMBRANE
GRO-α	GROA	CHEMOKINE	SURFACE

**Table 3:** Leukocyte receptors, their corresponding ligands, and their allowed behaviors

**Leukocyte Receptor**	**Surface Receptor-Ligand**	**Behaviors**
PSGL-1	P-Selectin	Rolling
VLA-4 Integrin	VCAM-1	Rolling, Firm Adhesion
CXCR-2 Chemokine Receptor	GRO-α Chemokine	Activation

These three receptor-ligand pairs were chosen because together they are sufficient for enabling leukocytes to roll, become activated, and firmly adhere in vitro.

**Table 4:** Model parameter values for the LEUKOCYTE MEMBRANE and LIGANDS along with corresponding in vitro values

**Parameter Name**	**Description**		**Model Parameter Value**	**Experimental Value**	**Reference**
Leuk*TotalWidth*	LEUKOCYTE MEMBRANE width (in the *y *[east-west] dimension)		20 MEMBRANE UNITS	Avg. Human Leukocyte Diameters (μm): Lymphocyte: 6.2; Neutrophil: 7; Monocyte: 7.5	[48]
Leuk*TotalLength*	LEUKOCYTE MEMBRANE length (in the *x *[north-south] dimension)		30 MEMBRANE UNITS		
Leuk*ExposedWidth*	CONTACT ZONE width (in the *y *dimension)		8 MEMBRANE UNITS	NA	NA
Leuk*ExposedLength*	CONTACT ZONE length (in the *x *dimension)		10 MEMBRANE UNITS	NA	NA
PSGL1*DensityMean *± PSGL1*DensitySTDev*	Mean number of PSGL-1 molecules (± SD) represented by each PSGL1 agent	Experiment 1: Neutrophils	150 ± 5^a ^molecules	~18,000/Human Neutrophil	[54]
		Experiment 3: Monocytes	150 ± 5^b ^molecules	N/A	N/A
VLA4*DensityMean *± VLA4*DensitySTDev*	Mean number of VLA-4 molecules (± SD) represented by each VLA4 agent	Experiment 2: T-lymphocytes	45 ± 5 molecules	3,000/Human T-Lymphocyte	[55]
		Experiment 3: Monocytes	60 ± 5 molecules	6,000/Human Monocyte	
CXCR2*DensityMean *± CXCR2*DensitySTDev*	Number of CXCR-2 molecules (± standard deviation) represented by each CXCR2 agent		1 ± 0 molecules	NA	NA
VLA4*MaxPercHighAff*	Maximum percent of VLA-4 integrins on the leukocyte membrane that can be induced into a high affinity state^c^		12.5%	10%	[49]
*Pon *(PSGL1-PSELECTIN1)	Probability of forming a PSGL1-PSELECTIN1 BOND		0.001	NA^d^	[56]
*Pon *(LOW AFFINITY VLA4-VCAM1)	Probability of forming a LOW AFFINITY VLA4-VCAM1 BOND		0.001	NA^d^	[55]
*Pon *(HIGH AFFINITY VLA4-VCAM1)	Probability of forming a HIGH AFFINITY VLA4-VCAM1 BOND		0.005	NA^d^	[55]
*Pon *(CXCR2-GROA)	Probability of forming a CXCR2-GROA BOND		1.0^c^	NA	NA
*Poff *(CXCR2-GROA)	Probability of breaking a CXCR2-GROA BOND		1.0^c^	NA	NA

^a ^PSGL1*DensityMean *± PSGL1*DensitySTDev *parameter values used for simulating Experiment 1, neutrophil rolling, were originally 25 ± 5. They were changed to the values currently listed to more closely reflect the experimental values of PSGL-1 density reported in the literature. No significant differences were observed when using these new parameter values.

^b ^Values for PSGL-1 sites/human monocyte were not found in the literature and were assumed to be similar to values reported for human neutrophils.

^c ^See Methods.

^d ^*Pon *and *Kon *are intended to map to aspects of the same in vitro phenomena. However, there is no direct mapping between these parameters because the parent models belong to fundamentally different classes [57].

**Table 5:** Experimental values for the three in vitro flow chamber environments and the corresponding parameter values used for each of the three simulated experimental conditions

	**In Vitro Values**			**ISWBC Values**		
**Experiment**	**Shear (dyn/cm^2^)**	**Substrate Molecule**	**Site density/Plating concentration**	***Rear Force***	**LIGAND**	**LIGAND Density**

**I. Neutrophil Rolling on P -Selectin**						
A. Pause Time	0.5, 1.0, 2.0	P-selectin	9 sites/μm^2^	0.1–0.5	PSELECTIN	15 ± 5
B. Distance-Time	2	P-selectin	25 sites/μm^2^	0.5	PSELECTIN	25 ± 5
C. Velocity-Time	0.5	P-selectin	9 sites/μm^2^	0.1	PSELECTIN	15 ± 5
**II. T-Lymphocyte Rolling on VCAM-1**						
Distance-Time	0.73 – 7.3	VCAM-1	15 μg/mL^a^	0.15–1.6	VCAM1	45 ± 5
**III. Monocyte Rolling on P-selectin/VCAM- 1/GRO-α**						
Rolling and Adhesion	1	VCAM-1	100 ng/mL^a^	1	VCAM1	13 ± 5
		P-Selectin	10 μg/mL^a^		PSELECTIN	25 ± 5
		GRO-α	5 μg/mL^a^		GROA	3 ± 2

The FLOW CHAMBER SURFACE dimensions were fixed at 100 × 60 SURFACE UNITS. Each SURFACE grid space maps to approximately 1 μm^2 ^of effective flow chamber surface area. ^a ^Values listed in units of [μg/mL] are the concentrations of the solutions of receptors used to coat the parallel plate flow chamber surfaces and are not meant to represent the concentrations of receptors found on the parallel plate surface.

**Table 6:** Calculated average ROLLING velocities, BONDS in the contact zone, and BONDS in the trailing row of the LEUKOCYTES' CONTACT ZONE

	***RearForce***				
	
	0.1	0.2	0.3	0.4	0.5
Average ROLLING velocity (μm/s)	3.42	5.19	7.98	10.68	14.16
Average number of BONDS in CONTACT ZONE	7.26	6.42	5.45	4.87	4.29
Average number of BONDS in the rear row of LEUKOCYTES' CONTACT ZONE	1.5	1.46	1.40	1.38	1.33

Averages are from 60 simulations that had at least 10 INTERACTIONS (pauses). ROLLING velocities fall within ranges reported in the literature. Average number of BONDS in the CONTACT ZONE is in agreement with values estimated by mathematical models, which range from two to twenty bonds
